# Mitf regulates gene expression networks implicated in B cell homeostasis, germinal center responses, and tolerance

**DOI:** 10.3389/fimmu.2024.1339325

**Published:** 2024-02-20

**Authors:** Abhimanyu Amarnani, Maria Lopez-Ocasio, Ramile Dilshat, Kamala Anumukonda, Jonathan Davila, Nikita Malakhov, Chongmin Huan, Erna Magnusdottir, Eirikur Steingrimsson, Christopher A. Roman

**Affiliations:** ^1^Program in Molecular and Cellular Biology, School of Graduate Studies, State University of New York (SUNY) Downstate Health Sciences University, Brooklyn, NY, United States; ^2^School of Medicine, State University of New York (SUNY) Downstate Health Sciences University, Brooklyn, NY, United States; ^3^Department of Medicine, Division of Rheumatology, New York University Langone Health, New York, NY, United States; ^4^National Heart, Lung, and Blood Institute, National Institutes of Health, Bethesda, MD, United States; ^5^Department of Biochemistry and Molecular Biology, Faculty of Medicine, Biomedical Center, University of Iceland, Reykjavik, Iceland; ^6^Anuko Tech Inc., Hillsborough, NJ, United States; ^7^Department of Urology, Northwell Health, Staten Island, NY, United States; ^8^Department of Hematology and Oncology, NewYork-Presbyterian-Weill Cornell Medical Center, New York, NY, United States; ^9^Department of Anatomy, Faculty of Medicine, Biomedical Center, University of Iceland, Reykjavik, Iceland

**Keywords:** B lymphocyte, tolerance, germinal center, transcriptome, microphthalmia transcription factor (Mitf), type-I-interferon

## Abstract

**Introduction:**

The *microphthalmia transcription factor* Mitf has been shown to regulate B cell activation and tolerance. However, the underlying B cell-specific mechanisms responsible, and those that distinguish Mitf from closely related Mitf/TFE (MiT) transcription factors Tfe3, Tfeb, and Tfec, remain obscure.

**Methods:**

Two complementary mouse models of Mitf and MiT deficiency were used: the Mitf^mi-vga9/mi-vga9^ systemic loss-of-function mutation, and B-cell specific MiT family inactivation via transgenic expression of a trans-dominant negative (TDN) protein (TDN-B). These models were employed to identify MiT family candidate target genes and pathways.

**Results:**

Both models displayed spontaneous splenomegaly coincident with elevated plasma cell numbers, autoantibody titers, and proteinuria. These abnormalities appeared dependent on T helper cells, but independent of other non-B cell intrinsic effects of systemic Mitf inactivation. MiT inactivation in B cells augmented aspects of lupus-like autoimmune disease on the C57BL/6-Fas^lpr/lpr^ background. In both models, RNAseq of *ex vivo* resting B cells showed transcriptional upregulation of genes that control cell cycle, germinal center responses, and plasma cell differentiation. Among the genes strongly upregulated in both models were Socs6, Isp53 (Baiap1), S1pR2, and IgG2b/c. Mitf null B cells, but not TDN-B cells, showed evidence of type I interferon dysregulation.

**Discussion:**

These studies clarify Mitf’s role as 1) a key regulator of a B cell intrinsic germinal center program that influences self-tolerance through novel target genes, and 2) a regulator of systemic inflammatory processes that can impact the B cell microenvironment. This distinction of Mitf's function from that of related MiT transcription factors advances our understanding of B cell regulation and autoimmunity.

## 1 Introduction

Mitf has been shown to play a role in mature B cell homeostasis and tolerance (1). Lin et al. (2005) showed that bone marrow chimeras, in which the grafted hematopoietic stem cells (HSCs) harbored a dominant negative mutation of Mitf (Mitf*^mi/mi^*), developed a lymphoid compartment in which a higher frequency of B cells was found to spontaneously differentiate into plasma cells in naïve mice. Also, these chimeric mice produced elevated levels of autoantibodies such as rheumatoid factor (RF) and anti-ssDNA (1). Complementary evidence from isolated Mitf*^mi/mi^* B cells in culture supported the conclusion that Mitf acted to restrain B cell activation by repression of Irf4, a key regulator of plasma cell development, given that in Mitf’s functional absence, Irf4 levels were elevated (1).

However, given that the Mitf*^mi/mi^* HSC transplantation model required a full reconstitution of all bone marrow derived cells by adoptive transfer into Rag-2-deficient mice, it remains unknown whether B cell specific loss of MiT family function leads to similar abnormalities. Furthermore, since the Mitf*^mi^* mutation encodes for a protein that lacks DNA binding ability, but still retains the ability to dimerize with all co-expressed MiT family members, dominant negative effects may contribute to those abnormalities (2). Thus, it is important to evaluate the effects of loss of Mitf alone and compare that to the situation where all MiT family function is blocked.

To address this, we compared the B cell compartments of two complementary mouse models for Mitf inactivation: a single gene model for B cell-specific MiT family inactivation (3), called TDN-B, in which a dominant negative inhibitor of the MiT family (4) is expressed in B cells only, and a germline recessive null Mitf mutation, Mitf^mi-vga9/mi-vga9^ (5) that cannot inhibit other MiT family proteins. With this strategy, we assessed whether TDN-B and Mitf^mi-vga9/mi-vga9^ mice presented with similar B cell abnormalities observed in the Mitf*^mi/mi^* HSC transplantation (HSCT) model (1). To gain further insight into how the loss of function of MiT molecules impacted control of B cell homeostasis and autoimmunity, we crossed the TDN-B gene onto the C57BL/6-Fas^lpr/lpr^ background and found enhancement of a subset of lupus-like autoimmune disease manifestations. A separate line of TDN transgenic mice, in which the TDN was expressed in both B and T cells (TDN-B/T) and has impaired T cell function as a result (4), was used to resolve B cell intrinsic abnormalities caused by Mitf inactivation from those dependent on T cell function. RNAseq analysis was performed on resting B cells from the different MiT/Mitf impaired strains to identify differentially regulated genes that could reveal underlying mechanisms that might explain the common and unique B cell abnormalities in each model. Our results show that inactivation of Mitf and the MiT family can impair B cell tolerance and perturb homeostasis mechanisms by derepressing a gene transcription program that regulates activation and germinal center responses.

## 2 Materials and methods

### 2.1 Mice

All mice were housed in specific pathogen-free conditions in the Division of Comparative Medicine at the State University of New York-Downstate Health Sciences University (SUNY Downstate); all experimental procedures were conducted according to protocols approved by its Institutional Animal Care and Use Committee. A recombinant gene encoding the trans dominant negative (TDN) protein was constructed from the TFE3 helix-loop-helix leucine zipper dimerization domain that lacks the DNA binding basic region, but was fused to a nuclear localization sequence and a hemagglutinin epitope (3, 4). The TDN-transgene contained the TDN cDNA subcloned into an immunoglobulin heavy-chain gene enhancer and promoter-based transgene cassette (E_µ_P_µ_) that directs expression in B cells (TDN-B) or both B and T cells (TDN-B/T) (3, 4)(**Supplementary Figure 1**). Mitf^mi-vga9/mi-vga9^ and Mitf^mi-vga9/+^ mice were a kind gift of the Arnheiter lab, and C57B/6-Fas^lpr/lpr^ were obtained from The Jackson Laboratory. To generate TDN-B.Fas^lpr/lpr^ mice, TDN-B animals (≥9 generations backcrossed TDN-B onto C57BL/6) were intercrossed with C57B/6-Fas^lpr/lpr^ animals. The presence of the TDN transgene was detected by PCR on tail or ear DNA using primers 5’-CACATAACAACCACATTCCTCCT-3’ and 5’-GAGCGTCCACAGGACCCTGAG-3’. Mitf^mi-vga9/mi-vga9^ mice were identified by their albino coat color. Mitf^mi-vga9/+^ were non-albino siblings of albino mice from litters of crosses between Mitf^mi-vga9/+^ females and Mitf^mi-vga9/mi-vga9^ males and spot checked by PCR detection of the insertion transgene (6). All TDN transgenic mice used for experiments were hemizygous for the TDN transgene; control mice were non-transgenic, sex-matched littermates of TDN-transgenic mice.

### 2.2 B cell enrichment

For RNA sequencing and *in vitro* culture studies, mouse B cells were enriched from splenocytes of 3-month-old, female mice using the Mouse B Cell EasySep Isolation Kit (StemCell Technologies, BC, Canada). Specifically, B cells were purified by negative selection against CD43, CD49b, CD90. 2, GR-1, TER119, CD11b, CD8a, and CD4, and including an FcR blocking antibody recognizing mouse CD16/CD32, to a purity of 96% or more as determined by flow cytometry. Purified B cells were counted by a TC-20 automated cell counter (Bio-Rad).

### 2.3 Flow cytometry and detection antibodies

For detection of cell surface markers, single-cell suspensions of splenocytes or bone marrow cells were treated with red blood cell (RBC) lysis buffer to eliminate erythrocytes and then stained following standard protocols. The following antibodies to characterize and enumerate B cells were used: CD19, CD21, CD23, IgM, IgD, CD69, CD25, CD95, CD38, CD138, Ly6A/E, Ly6C, AnnexinV, I-A[b], and CD86. Markers for T cell studies were: CD4, CCR7, CD5, CD54, CD279, CD62L, and CD44. Antibodies were conjugated to FITC-, PE-, PerCP-Cy5.5-, PE-Cy7-, or APC-Cy7, and were purchased from eBiosciences or BD Biosciences. Data acquisition was performed on a FACScan (BD Biosciences) or Guava easyCyte flow cytometer (EMD Millipore) and analyzed by CellQuest or FlowJo software (v10.0.07). Live vs. dead cells were distinguished on the Guava EasyCyte using the Live/Dead Fixable Far Red Dead Cell Stain kit (ThermoFisher Scientific). Lymphocyte gate was determined by forward scatter vs. side scatter properties.

### 2.4 Immunizations

To evaluate thymus-independent (TI) responses to a model type II antigen, ~8-month-old wild-type, TDN-B and TDN-B/T mice were immunized intraperitoneally with 20 µg of the trinitrophenol (TNP) conjugated to Ficoll (Biosearch Technologies) in 1x PBS. Mice were bled right before and 7 d after immunization.

### 2.5 Enzyme-linked immunosorbent assays

Enzyme-linked immunosorbent assay (ELISA) studies were completed to measure total, autoimmune, and TNP-specific antibodies. In brief, 96-well plates were bound by anti-IgM or anti-IgG antibodies at 10ug/mL, at room temperature (RT) incubation for 1 hour. Plates were washed with 1x TBS-Tween20 (TBS-T) 5 times. Blocking buffer was then added (1% BSA in 1X TBS) to each well to block any non-specific binding sites on the surface and incubated for one hour at RT. Sera were diluted for IgM at 1:10,000 and for IgG at 1:50,000. Plates were then washed 5 times with TBS-T. HRP antibody against either IgM or IgG was diluted with blocking buffer followed by a series of dilutions, followed by incubation for one hour. Plates were washed again with 1X TBST. TMB was then added to develop positive signal (blue color). The color reaction was stopped with a 1:10 dilution of 1M H2SO4 and read immediately in a plate reader at 450nm.

For autoantibody assays, serum levels of IgM and IgG anti-ssDNA and anti-dsDNA were detected by incubating serum diluted 1:100 into poly-L-lysine plates precoated with either ssDNA or dsDNA, as described (7). Detection antibodies were IgM-HRP and IgG-HRP. Serum anti-chromatin antibodies were detected with chicken chromatin, as described (7).

TNP titers were measured in Immuron plates (Dynex) coated with TNP-bovine serum albumin (BSA) (Biosearch Technologies) using HRP conjugated ELISA detection antibodies (Bethyl). Plates were incubated with 10 µg/mL of coating antibody (Bethel Laboratories). Sera were diluted in blocking buffer at 1:10,000 for IgM and 1:10,000 for IgG. For IgG isotypes, the same protocol was followed with a variation in the concentration of the isotypes: 1:40,000 for IgG2b and IgG2c.

### 2.6 B cell culture and supernatant multiplex assays

Purified B cells were cultured in RPMI medium (Hyclone) supplemented with 10% heat-inactivated FBS (Corning), 1% L-Glutamate (w/v), 1% Streptomycin and Ampicillin (w/v), and 55µm β-mercaptoethanol (Sigma), at a density of 2-2.5 million cells/mL. For stimulation, purified mouse splenic B cells were cultured with lipopolysaccharide (25µM/mL, *Salmonella enterica* serotype typhimurium) or IgM (10µg/mL) and CD40L (1µg/mL) for 20-24hrs. The abundance of cytokines in culture supernatants was measured using the Mouse Cytokine/Chemokine MILLIPLEX MAP multiplex kit (EMD Millipore, Burlington, MA) with a dual laser, flow-based multiplex assay (Luminex 200 TM, Luminex Corporation, Austin, TX) allowing simultaneous quantification of IL-10 and tumor necrosis factor-alpha in single samples. All sample assays were carried out in triplicate and averaged to generate a value for each biological replicate.

### 2.7 RNA-sequencing and analysis

For RNA-sequencing (RNA-seq) of highly enriched ex-vivo B cells, between 1 and 5 million cells were disrupted in Trizol Reagent and samples shipped on ice overnight to the laboratory of ES at the University of Iceland Biomedical Center. Isolation of mRNA from total RNA was accomplished with magnetic beads conjugated to oligo dT; then, RNA fragmentation, random priming, cDNA synthesis, adaptor ligation, and PCR enrichment was completed, using the NEBNext Ultra Directional RNA Library Prep Kit for Illumina. A bioanalyzer (Agilent), was used as an RNA-seq library preparation quality check. 30 million reads per sample, with a read length of ~2x125 bp, were sequenced by paired end sequencing, and then aligned to the *Mus musculus* transcriptome by Kallisto (8), utilizing over 45,000 transcripts evaluated that passed filtering quality control in Sleuth (9). In each of the samples analyzed, expression was normalized as transcript per million. Gene expression results were displayed in the Perseus platform (10). Differential gene expression analysis was calculated with Sleuth (9) in R. Significance was defined by q value < 0.05 based on the likelihood ratio test for comparison of *transcript abundance among models.* The likelihood ratio does not produce a metric equivalent to the fold change, rather, the Wald test is used to define differential expression between each sample pairing. The Wald test beta statistic approximates the log2 fold change in expression between two conditions tested. The likelihood ratio is considered the statistical test with fewer false positives compared to the Wald test, thus, significance filtering is based on likelihood q values. Beta values (natural log2 with technical variation removed) was converted to log2 fold change using the formula LOG(POWER(2.71828, (Beta Value)), 2) in Excel. Pathway enrichment analysis was completed through Enrichr (11). ChEA3 (12) was utilized for transcription factor network analysis with results output as “Top Rank,” indicating the top integrated rank across libraries.

### 2.8 Real time RT-PCR

Total RNA from approximately 9 million purified splenic B cells per time point were analyzed via real time RT-PCR. RNA was isolated with Trizol Reagent (Ambion) and Direct-zol RNA mini-prep plus kit (Zymo) according to the manufacturer’s instructions. Total RNA was reverse-transcribed by Verso cDNA synthesis Kit (Thermo Scientific) according to the manufacturer’s instructions with a 3:1 blend of random hexamers and anchored oligo-dT primers for first-strand synthesis. cDNA was amplified by real time-PCR with the Maxima SYBR Green qPCR Master Mix (Thermo Scientific) in a CFX384 Touch Real-Time PCR Detection System (Bio-Rad). All RT-qPCR experiments were completed in triplicate. The relative abundance of mouse cDNA expression was measured relative to β-Actin. PCR analysis Data Collection, Data Analysis, and Statistics were completed with the CFX Maestro Software (Bio-Rad). All primers used had an efficiency greater than 90% and a delta-delta Ct was used to assess relative qPCR amplification between samples.

### 2.9 Immunostaining

Spleen sections were snap-frozen embedded in OCT (Tissue Tek) and stored at -80C until sectioning. 14µM thick sections on slides were fixed in Acetone for 10 minutes. Slides were then removed from the fixation agent and dried for 10 min, and then washed three times with PBS+0.1%Tween-20 (PBS-T) and incubated for 1hr with blocking solution containing 5% normal goat serum and 0.3%Triton x-100 diluted in PBS-T. After the blocking solution was removed, the primary antibody was applied to the tissue section overnight at 4°C, in a humid chamber, at the following dilutions: anti-B220 (primary 1:250 dilution, abcam, RA3-6B2), anti-CD3 (1:200, abcam, ab5690). After three PBS-T washes, tissue sections were then incubated with secondary antibody for 2h at room temperature, at the following dilutions: anti-rat Alexa488 (1:100, abcam, ab150165), anti-rabbit Alexa594 (1:200, abcam, ab150084). Sections were again washed three times in PBS-T and then either incubated with counterstain, or next mounted with Hardset Antifade Mounting Medium (Vecta Shield) and covered with a coverslip. Sections were viewed and imaged by wide-field, compound fluorescence microscopy (Axio Vision 4.7, Carl Zeiss), and/or confocal microscopy (Leica TCS SP-5). Immunoreactivity was measured using FIJI ImageJ software program (http://fiji.sc/Fiji). For fluorescence staining, splenocytes were smeared onto a poly-L-lysine coated slide (Fisherbrand, Plus), fixed with 4% Formaldehyde for 15 minutes at room temperature, and next fixed with 100% ice-cold methanol for 10minutes in a -20C freezer. Blocking, staining, washing, and mounting were completed as indicated above.

### 2.10 Statistical analyses

Kruskal-Wallis test was used with multiple comparisons *post-hoc* to analyze flow cytometry and other biometric data. Error bars in figures are standard error of the mean. Experiments were completed with n ≥ 3 biological replicates per group. For RNA sequencing analyses, after Kallisto alignment and filtering, normalized data was completed through Sleuth (9). Significant differential expression was determined through the likelihood goodness-of-fit model comparing wild type gene expression (null model) against the Mitf and MiT family functional impairment sample gene expression (alternative model) at a threshold of q-value (adjusted p-value) <0.05. For pathway enrichment, significantly enriched pathways were assessed through Fisher’s exact test at an adjusted p value (qval) cut off < 0.05. Data analysis was graphed with Prism 7 or 8 (GraphPad) software or Perseus (10). Meta-analysis of microarray data from a prior study (13) of human B cells utilizes significance analysis of microarray (SAM) multiclass analysis, unpaired Wilcoxon statistics, with a fold change ≥ 2 to define significance of differential expression.

## 3 Results

### 3.1 Systemic (germline) inactivation of Mitf or B-cell-specific MiT lead to spontaneous abnormalities of the mature B cell compartment

Original studies revealing a role of Mitf in regulating B cell function used a bone marrow chimera model in which HSCs from the Mitf*^mi/mi^* mouse strain were engrafted to Rag-2 deficient recipients (1). This strategy was necessitated because of the severe osteopetrosis caused by the *Mitf^mi^* mutation, which leads to lethality 3 weeks after birth. The Mitf^mi^ mutant protein is dominant negative and inactivates Tfe3, which acts redundantly with Mitf during osteoclastogenesis (14). In the engrafted chimeras, a higher frequency of Mitf*^mi/mi^* B cells were found to spontaneously differentiate into plasma cells. However, given that the entire hematopoietic system was derived from Mitf*^mi/mi^* mice, it was unclear whether a loss of MiT function only in B cells would yield a similar phenotype. Myeloid lineage cells and dendritic cells are known to influence B lymphocytes, and their properties in this capacity could have been influenced by the dominant negative Mitf mutation (15–17). Similarly, given the dominant negative property of the mutant Mitf protein produced by the *Mitf^mi^* allele, it was unclear whether a strict loss-of-function Mitf mutation by itself would yield a similar B cell phenotype.

We utilized a previously described transgenic approach (4) to express a recombinant *trans* dominant negative (TDN) protein specific for the MiT family in lymphocytes. The TDN protein contains the Tfe3 helix-loop-helix-leucine zipper dimerization domains, but lacks the DNA-binding basic region and transcription activation domains. This property, along with engineered nuclear localization sequences, allows it to form inactive heterodimers with endogenous MiT proteins that are incapable of binding DNA (3). We first focused on one line, TDN-B, in which the TDN is expressed exclusively in the B lineage (**Supplementary Figure 1**, See section 2.1), as a model for B-cell specific MiT inactivation. In parallel, we evaluated a mouse homozygous for the recessive loss-of-function (“null”) allele Mitf^mi-vga9^ (5). These mice have a white coat and severe microphthalmia due to the requirement of Mitf for the development of both melanocytes and retinal pigment epithelial cells. However, unlike Mitf*^mi/mi^*, these mice do not have osteopetrosis because Tfe3, redundantly required for osteoclast development, is not inhibited.

**Figure 1:**
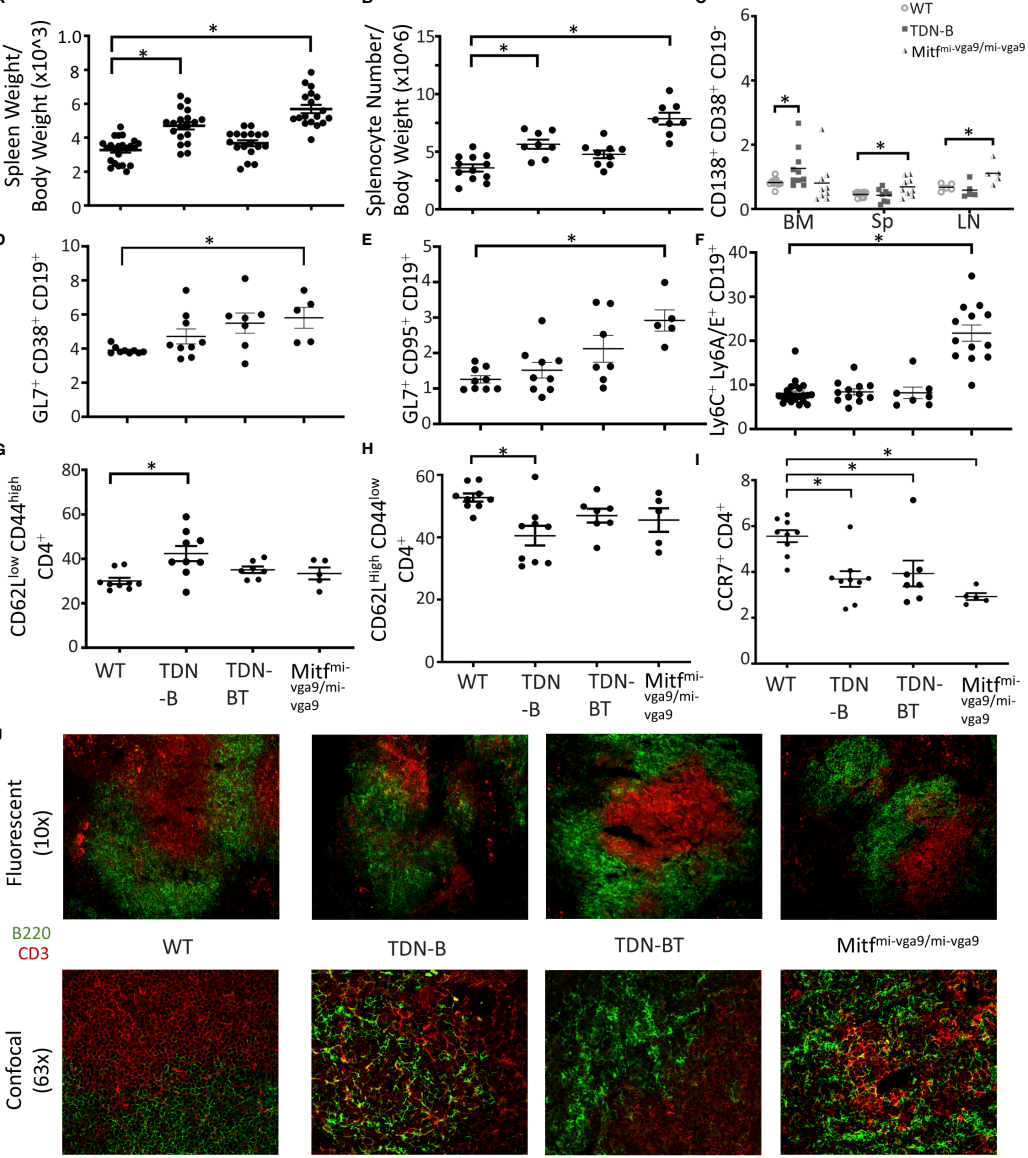
Altered lymphoid compartments and lymphocyte subpopulations in Mitf and/or MiT/TFE deficient mice. **(A, B)** Spleen weight and splenocyte number relative to body weight of wildtype, TDN-B, TDN-BT, and Mitf^mi-vga9/mi-vga9^ mice. 2-4 months old. **(C)** Frequency of plasma cells in bone marrow (BM), spleen (Sp), and lymph nodes (LN) of wildtype, TDN-B, and Mitf^mi-vga9/mi-vga9^ mice. 3-6 months old. **(D, E)** Relative frequencies of multipotent germinal center precursor cells **(D)** and germinal center B cells **(E)** in wildtype, TDN-B, TDN-BT, and Mitf^mi-vga9/mi-vga9^ splenocytes. 3-6 months old. **(F)** Lymphocyte antigen-6A/E family protein positive B cells in wildtype, TDN-B, TDN-BT, and Mitf^mi-vga9/mi-vga9^ splenocytes. 10-12 months old. **(G)** Active effector (CD62LlowCD44high) helper T cell, **(H)** naïve helper (CD62LhighCD44low) T cell, and **(I)** C-C chemokine receptor type 7 helper T cell, and proportions of wildtype, TDN-B, TDN-BT, and Mitf^mi-vga9/mi-vga9^ splenocytes. 4-7 months old. Kruskal-wallis with multiple comparisons *post-hoc*, error bars indicate SEM, *p<0.05. **(J)** Microscopy of splenic primary follicles at low magnification wide-field microscopy (top) and confocal microscopy (bottom). CD3 is shown in red, B220 is shown in green. Representative of at least three experiments; 6-8 months old. All data shown age and sex matched, male and female mice.

Both TDN-B and Mitf^mi-vga9/mi-vga9^ mice exhibited spontaneous development of splenomegaly, evident starting at 3 months (**Figure 1A**), which correlated with a roughly proportional increase in total splenocyte numbers (**Figure 1B**). Within this expanded population, the relative proportion of CD19+ cells appear unchanged (**Supplementary Figures 1**). In addition, B cell developmental stages in the bone marrow and spleen were largely intact, with a subtle increase in the proportion of pre-B cells and decrease in the proportion of marginal zone B cells noted in TDN-B mice (**Supplementary Figures 2**–**4**)]. However, flow cytometry showed that the percentages of cells with markers consistent with antibody secreting plasma cells (CD19^–^CD38+CD138+) were elevated in both TDN-B and Mitf^mi-vga9/mi-vga9^ mice when compared to controls (**Figure 1C**). This was most evident in Mitf^mi-vga9/mi-vga9^ spleens and lymph nodes, and the bone marrow of TDN-B mice (**Figure 1C**). Multipotent germinal center precursor B cells (18) (CD19+GL7+CD38+) and germinal center B cells (CD19+CD95+GL7+) were significantly increased in Mitf^mi-vga9/mi-vga9^ mice spleens (**Figures 1D, E**).

Recent studies on the consequences of Mitf haplo-insufficiency on melanocyte progenitor cells have suggested that global Mitf deficiency dysregulates innate immune responses systemically, leading to elevated expression of type I interferon (IFN) genes (19). Elevated type I IFN is also a characteristic of lupus autoimmunity (20). To determine whether the B cell abnormalities observed in both models were a reflection of that generalized defect, we analyzed lymphocytes for expression of markers known to be upregulated by type I IFNs (19, 21). Interestingly, B cells from Mitf^mi-vga9/mi-vga9^ mice had an increased frequency of CD19+ B cells expressing surface Ly6C and Ly6A/E compared to controls (**Figure 1F**). However, TDN-B transgenic B cells were indistinguishable from non-transgenic controls and showed no evidence of this change. We therefore conclude that ostensibly resting B cells from Mitf^mi-vga9/mi-vga9^ mice indeed express markers consistent with a deregulation of type I IFN responsive genes, and that these abnormalities do not contribute to splenic B cell compartment abnormalities in TDN-B mice. Thus, the impact of Mitf inactivation on B cells is distinct from the generalized systemic defect in type I IFN homeostasis.

Coincident with splenomegaly, spleens from naive TDN-B and Mitf^mi-vga9/mi-vga9^ mice also contained many follicles that appeared disorganized, characterized by the lack of discrete delineation between the peri-arteriolar lymphoid sheath and follicles, with confocal microscopy showing CD3+ cells within B cell (B220+) areas (**Figure 1J**). This led us to analyze splenic CD4+T cells by flow cytometry. This revealed some subtle differences in the Mitf-null and MiT-inactivated strains, with differences from control mice not always being identical to each other. For example, in TDN-B, but not Mitf^mi-vga9/mi-vga9^ spleens, the fraction of CD62L^low^CD44^hi^ CD4+ cells, indicative of effector helper T cells, was elevated (**Figures 1G, H**; **Supplementary Figure 2D**), whereas in all Mitf-deficient models(null and inactivated) the fraction of CD4^+^ cells expressing the homing molecule CCR7 was decreased (**Figure 1I**). In contrast, Mitf^mi-vga9/mi-vga9^ spleens had a reduced frequency of CD279(PD-1)^+^CD4^+^ T cells, with TDN-B spleens trending similarly (**Supplementary Figure 2I**). The frequency of CD54(ICAM-1)^+^CD4^+^T cells was not different among groups (**Supplementary Figure 2J**). There were no significant changes in B cell compartments for immature, mature, recirculating, or T1, T2, and T3 B cells (**Supplementary Figures 3**, **4**). These data indicate that CD4+T cells were responding to changes caused by the inactivation of MiT family members in B cells, but also were affected by non-B cell autonomous functions driven by unknown effects of Mitf loss-of-function or MiT inactivation in non-B cells in each model.

### 3.2 Spontaneous B cell abnormalities in TDN-B mice are influenced by helper T cells

To explore whether T-cell help is required for the B cell abnormalities in TDN-B mice, we analyzed a different TDN line, TDN-B/T, in which the TDN transgene was expressed in both B and T cells. As we have reported, TDN expression exclusively in T cells impairs helper CD4 T cell function by reducing expression of CD40L, which is regulated jointly by Tfe3 and Tfeb (4). In the TDN-B/T model, splenocyte cellularity and mass (**Figures 1A, B**), and its follicular architecture (**Figure 1J**), were virtually indistinguishable from controls. Of note, the splenic compartment in TDN-B/T mice still contained significantly decreased CCR7+CD4+ T cells similar to TDN-B and Mitf^mi-vga9/mi-vga9^ spleens (**Figure 1I**). Overall, these observations suggest that B cell intrinsic drivers are responsible for splenic B and T cell subset changes.

### 3.3 MiT family inactivation in B cells selectively influences spontaneous and induced antibody titers

**Figure 2:**
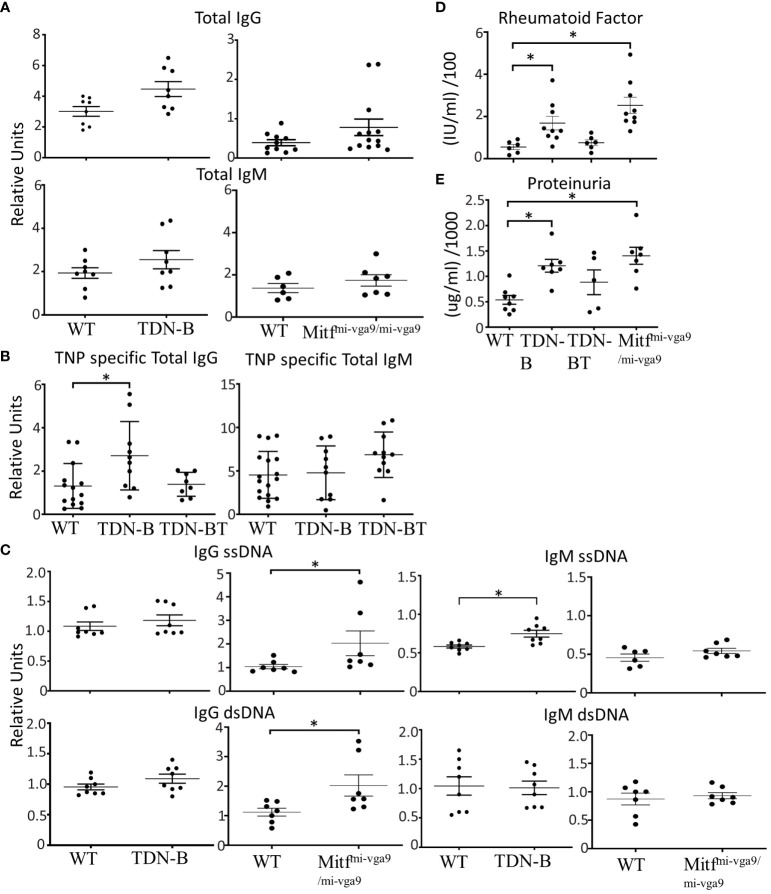
Selective enhancement of antibody and autoantibody titers and proteinuria in mice with Mitf and/or MiT/TFE family deficiency. **(A)** Total IgG and Total IgM titers detected in peripheral blood of unimmunized wildtype, TDN-B, and Mitf^mi-vga9/mi-vga9^ mice. (left) 6-7 months old (right) 5-8 months old mice. **(B)** 2,4,6-Trinitrophenyl hapten-FICOLL specific (T independent) total IgG and total IgM detected in peripheral blood seven days post-immunization of wildtype, TDN-B, TDN-BT, and Mitf^mi-vga9/mi-vga9^ mice. 8-10 months old. **(C)** IgG ssDNA, IgG dsDNA, IgM ssDNA, and IgM dsDNA titers detected in peripheral blood of unimmunized wildtype, TDN-B, and Mitf^mi-vga9/mi-vga9^ mice. Related OD reading units OD450. WT vs. TDN-B 6-7 months old. WT vs. Mitf^mi-vga9/mi-vga9^. 5-8 months old. **(D)** Serum rheumatoid factor titers in wildtype, TDN-B, TDN-BT, and Mitf^mi-vga9/mi-vga9^ peripheral blood. 5-8 months old. **(E)** Protein detected by colorimetric assay of urine from wildtype, TDN-B, TDN-BT, and Mitf^mi-vga9/mi-vga9^ mice. 5-8 months old. Kruskal-wallis w/multiple comparisons *post-hoc*, error bars indicate SEM, *p<0.05. All data shown age and sex matched, male and female mice.

Given the spontaneous B cell abnormalities in TDN-B and Mitf^mi-vga9/mi-vga9^ mice, we measured serum antibody levels. Since serum from unimmunized TDN-B and Mitf^mi-vga9/mi-vga9^ mice showed no significant difference in IgG or IgM levels compared to controls (**Figure 2A**), we measured levels of induced antigen-specific antibody produced because of immunization. Given the possible confounding effects of Mitf-deficiency on other cells that are involved in vaccine responses, we focused the assessment on TDN-B mice, and immunized them using a standard thymus-independent (TNP-Ficoll) vaccine protocol. At day 7 post-TNP-Ficoll immunization, anti-TNP IgM in serum from TDN-B mice was the same as controls and TDN-B/T mice, but total anti-TNP IgG levels were elevated in TDN-B mice compared to controls and TDN-B/T mice (**Figure 2B**) Therefore, the helper T cell -dependent changes due to Mitf and MiT family dysfunction include a modest and selective enhancement of induced and spontaneous antibody production.

### 3.4 MiT inactivation augments aspects of lupus-like disease in Fas*^lpr/lpr^* mice

Mitf *^mi/mi^* HSC chimeric mice were reported to have elevated levels of anti-ssDNA and RF (1). Analysis of TDN-B and Mitf^mi-vga9/mi-vga9^ sera showed similarly modest elevations of these autoantibody titers (**Figures 2C, D**). Also consistent with the HSCT model, both strains had elevated titers of rheumatoid factor (**Figure 2D**), which appeared to be helper T cell dependent, as this was not evident in the TDN-B/T strain. Like the HSCT model, 6-8 month old TDN-B mice had elevated levels of anti-ssDNA IgM, whereas Mitf^mi-vga9/mi-vga9^ sera contained elevated anti-dsDNA IgG (**Figure 2C**). Both TDN-B and Mitf^mi-vga9/mi-vga9^ mice had increased proteinuria compared to WT and B/T mice (**Figure 2E**), which correlated with rheumatoid factor levels (**Figure 2D**). Thus, the type of elevated autoantibody production was similar in both strains and paralleled what was observed in the Lin et al (1). study. **Supplementary Figure 5** summarizes phenotypes and B cell abnormalities in TDN-B and Mitf^mi-vga9/mi-vga9^ mice.

**Figure 3:**
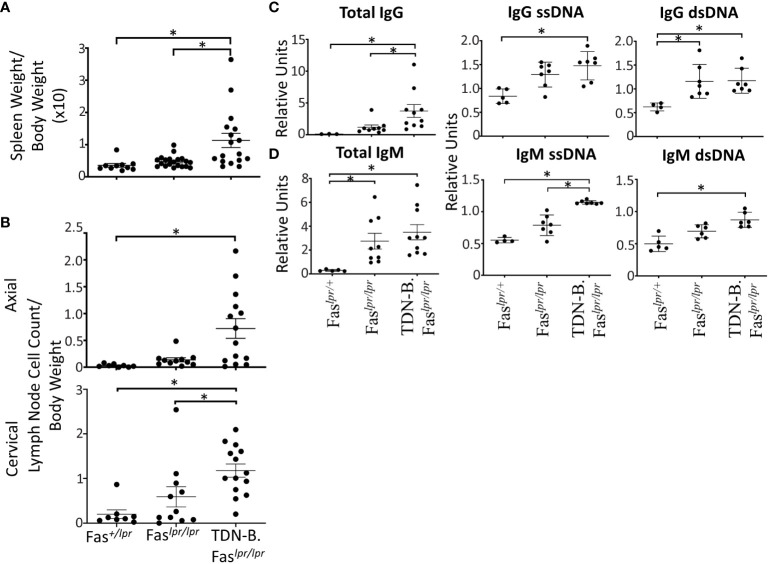
Autoantibody production, splenomegaly, lymphadenopathy, and renal damage caused by the lpr mutation is augmented by B cell specific MiT family inactivation. **(A)** Splenomegaly and **(B)** lymphadenopathy in Fas^+/lpr^ (control), Fas*^lpr/lpr^*, and TDN-B.Fas*^lpr/lpr^* mice. 4.5-7.5 months old. **(C)** Total IgG, IgG ssDNA, IgG dsDNA, **(D)** total IgM, IgM ssDNA, IgM dsDNA dsDNA titers detected in Fas^+/lpr^, Fas*^lpr/lpr^*, and TDN-B.Fas*^lpr/lpr^* peripheral blood. 4.5-7.5 months old. Kruskal-wallis w/multiple comparisons *post-hoc*, error bars indicate SEM, *p<0.05. 4-6 months old, Mann-whitney U, *p<0.05. All data shown age and sex matched, male and female mice.

As another way to assess the significance of the autoimmune related phenomena, we asked whether Mitf/MiT family inactivation in B cells could influence the characteristics of autoimmunity and autoimmune disease in the established C57BL/6-Fas^lpr/lpr^ model for mild lupus. To accomplish this, the TDN-B transgene was crossed onto the C57BL/6-Fas*^lpr/lpr^* background, creating the TDN-B.Fas*^lpr/lpr^* mouse line. Indeed, TDN-B.Fas*^lpr/lpr^* mice appeared to have more pronounced lymphadenopathy and splenomegaly (**Figures 3A, B**; **Supplementary Figure 6A**), and elevated autoantibody titers to certain autoantigens (**Figures 3C, D**), compared to matched Fas*^lpr/lpr^* mice. Enumeration of total cells recovered from the axillary and superficial cervical lymph nodes indicated greater numbers per body mass in the TDN-B.Fas*^lpr/lpr^* cohort compared to matched Fas*^lpr/lpr^* mice (**Figure 3B**). However, flow cytometry analysis showed that the fraction of lymphocytes and B cells were similar (**Supplementary Figure 6B**), suggesting that MiT inactivation did not further amplify the known effects of the lpr mutation. Neither splenomegaly nor lymphadenopathy were observed in heterozygote C57Bl/6-Fas^lpr^/+ mice (Fas^lpr/+^) at 6 months of age (**Figures 3A, B**).

With respect to autoantibodies, total IgG and IgM ssDNA were significantly increased in TDN-B.Fas*^lpr/lpr^* mice compared to both wild type and Fas*^lpr/lpr^* mice (**Figures 3C, D**), while IgG ssDNA and IgM dsDNA where significantly increased compared to wild type in sera of TDN-B.Fas*^lpr/lpr^* mice but not Fas*^lpr/lpr^*. In contrast, no significant difference in anti-chromatin antibody titers was observed (**Supplementary Figure 6C**), indicating some selectively in the range of auto-specificities affected by loss of Mitf/MiT function.

### 3.5 Quantitative transcriptomics of B cells with functional impairments of Mitf and the MiT family transcription factor family

**Figure 4:**
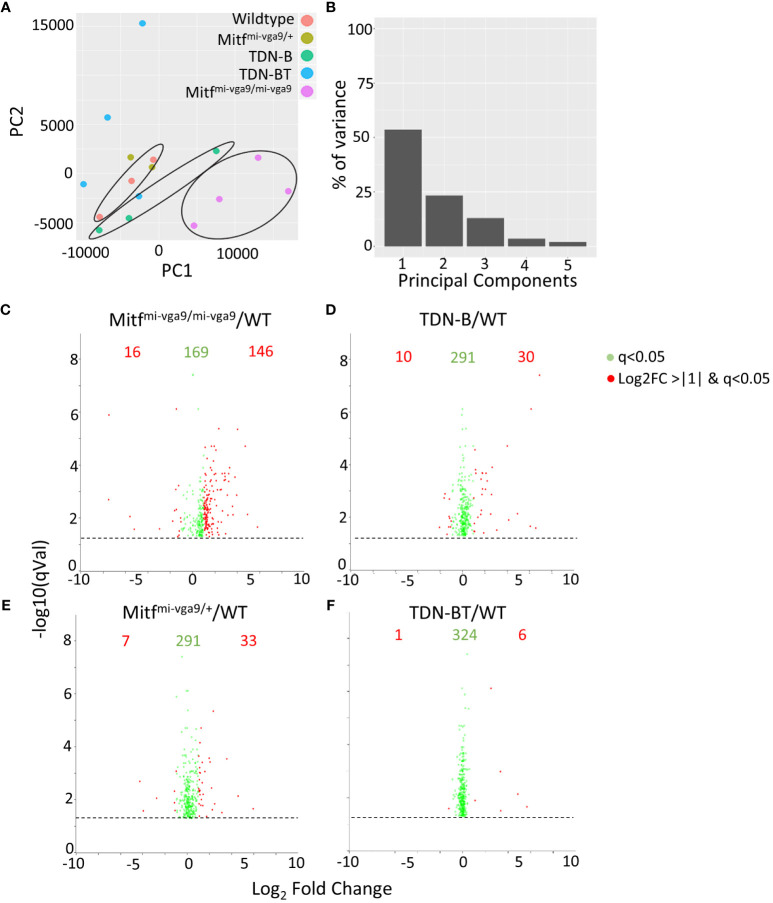
Gene expression intensities in B cells with Mitf and/or MiT/TFE family functional impairment. **(A)** Principal component analysis (PCA) of normalized transcripts per million data from Mitf^mi-vga9/mi-vga9^ (pink), TDN-B (green), TDN-B/T (blue), Mitf^mi-vga9/+^ (gold), and wildtype (red) B cells. **(B)** Percentage of transcript variance in PCA determined by PC1, 2, 3, 4, and 5. **(C–F)** Pairwise comparison of quantification ratios between B cells of wildtype, TDN-B, TDN-BT, Mitf^mi-vga9/+^, Mitf^mi-vga9/mi-vga9^ B cells. Shows significantly differentially expressed transcripts, likelihood ratio q<0.05. Red transcripts indicate those quantified >+1 or <-1 with a log_2_ratio. Data shown 3 months old, female mouse purified B cells.

To get a better understanding of how the absence of Mitf and MiT family function impacted the transcriptomic landscape in a way that could explain these abnormalities, we performed RNA-sequencing of ostensibly resting mature B cells isolated through negative selection from wild-type control, Mitf^mi-vga9/mi-vga9^ and TDN-B mice. We also included an analysis of B cells from mice heterozygous for the Mitf null mutation (Mitf^mi-vga9/+^) as a way to explore Mitf gene dosage effects on B cell target genes, given the impact of Mitf heterozygosity on premelanocytes (19). Finally, we included B cells from TDN-B/T mice in the analysis to explore the involvement of T cell help in shaping the MiT-deficient transcriptomic landscape. A principal component analysis (PCA) of gene expression was used to summarize the variance of data among the models and plot how each transcript covaries across the samples (**Figures 4A, B**). This PCA was completed based on purified B cell samples from the five genotypes studied and shows segregation of wild type, TDN-B and Mitf^mi-vga9/mi-vga9^ samples. **Supplementary Table 1**, shares the likelihood ratio adjusted p values (q value) (9), gene names, uniprot accession numbers (22), log_2_fold change of each model’s gene expression data against wild type, and Ensembl annotations (23) of all transcripts across samples. **Supplementary Table 2** provides a summary of all five mouse models utilized in RNA-Seq experiments. Using the likelihood ratio for significance filtering cut off, 331 transcripts are significantly differentially expressed (q<0.05), comparing transcript abundance across all the models (9). When comparing MiT functional impairment and wild type B cells, a subset of these transcripts was differentially expressed with a log_2_ fold change of <-1 or >+1. The significant cut off was defined by the likelihood ratio q<0.05 among all models, and the fold change is determined by the Wald test between each pairwise comparison (**Figures 4C–F**, indicated in red).

**Figure 5:**
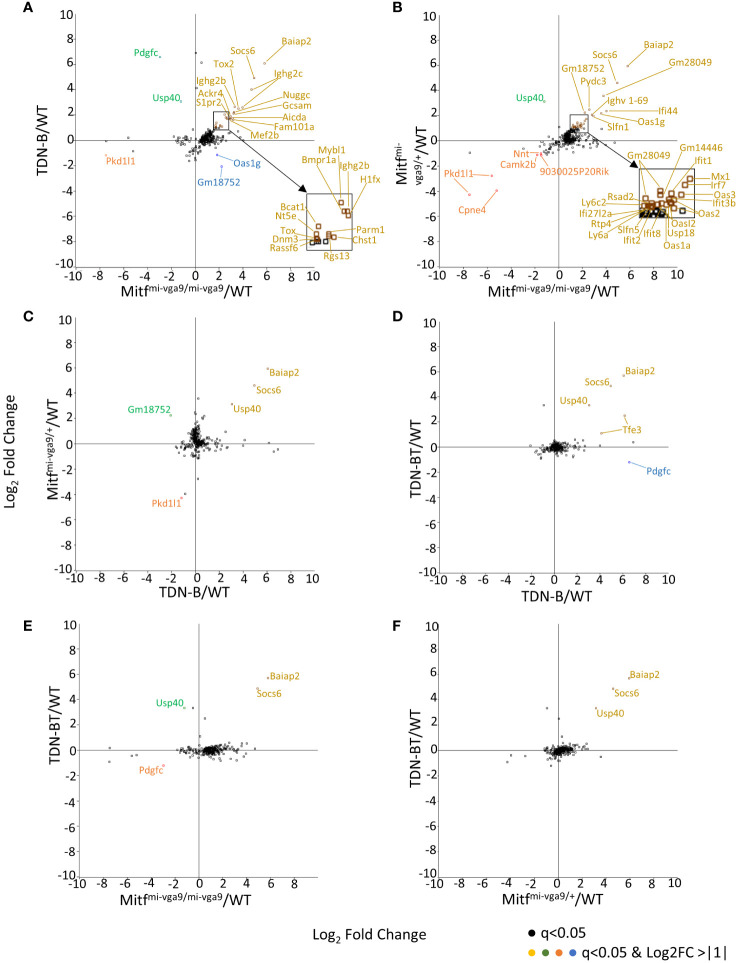
Pairwise comparison of transcripts with high magnitude differential expression in models of Mitf and/or MiT/TFE family functional impairment. **(A–F)** Scatterplots of transcripts with log2ratio transcript expression of >1 or <-1 of Mitf and MiT/TFE family functional impairment models compared to wildtype. **(A)** TDN-B vs. Mitf*^mi-vga9/mi-vga9^*, **(B)** Mitf^mi-vga9/+^ vs. Mitf*^mi-vga9/mi-vga9^*, **(C)** Mitf^mi-vga9/+^ vs. TDN-B, **(D)** TDN-BT vs. TDN-B, **(E)** TDN-BT vs. Mitf*^mi-vga9/mi-vga9^*, **(F)** TDN-BT vs. Mitf*^mi-vga9/mi-vga9^*. All transcripts are colored as follows (X,Y): Gold = (+,+), Blue = (+,-), Orange = (-,-), Green = (-,+). 3 months old. 3 months old female mouse purified B cells.

The 331 transcripts that meet the likelihood ratio significance cut off (q<0.05) were plotted to compare relative expression of transcripts across TDN-B, TDN-B/T, Mitf^mi-vga9/mi-vga9^, and Mitf^mi-vga9/+^ models against wild type expression (**Figures 5A–F**). **Figure 5A** provides a scatterplot of significantly differentially expressed transcripts, plotting relative expression of TDN-B against Mitf^mi-vga9/mi-vga9^ B cells (**Figure 5A**). Colored and labeled transcripts are those that also have a log2 fold change greater than one. Relative expression in Mitf^mi-vga9/+^ against Mitf^mi-vga9/mi-vga9^ (**Figure 5B**), Mitf^mi-vga9/+^ against TDN-B (**Figure 5C**), TDN-B/T against TDN-B (**Figure 5D**), TDN-B/T against Mitf^mi-vga9/mi-vga9^ (**Figure 5E**), and TDN-B/T against Mitf^mi-vga9/mi-vga9^ (**Figure 5F**) are also plotted. The expression of genes known to be involved with germinal center organization and development were shown to be significantly increased in both TDN-B and Mitf^mi-vga9/mi-vga9^ B cells and include Aicda (24), Rgs13 (25), S1pr2 (26), Mef2b (27), and Nuggc (28) (**Figure 5A**). In contrast, genes shown to be induced in early plasma cells (13) (Oas3 and Oasl2) and plasmablasts (13) (Irf7, Ifit1, ifi44), as well as those related to type I interferon responses, were shown to be significantly increased in both Mitf^mi-vga9/+^ and Mitf^mi-vga9/mi-vga9^ B cells (**Figure 5B**), but not in TDN-B B cells. **Table 1** highlights 25 transcripts differentially expressed in both TDN-B and and Mitf^mi-vga9/mi-vga9^ B cells, including many previously identified as important for germinal center function. Few transcripts were differentially expressed in B cells of the other MiT functional deficiency models when compared to wild type. Only significantly differentially expressed transcripts, defined by their likelihood ratio q<0.05, are indicated in **Figure 5**.

**Table 1:** Summary of differentially expressed transcripts.

A				
Upregulated Genes (q<0.05 & Log2>1)	Mitf^mi-vga9/mi-vga9^	TDN-B	Mitf^mi-vga9/+^	TDN-BT
Baiap2	x	x	x	x
Socs6	x	x	x	x
Ighg2c	x	x		
Gcsam	x	x		
Tox2	x	x		
Nuggc	x	x		
Fam101a	x	x		
Aicda	x	x		
Ighg2b	x	x		
Ackr4	x	x		
Mef2b	x	x		
S1pr2	x	x		
H1fx	x	x		
Bmpr1a	x	x		
Mybl1	x	x		
Rgs13	x	x		
Chst1	x	x		
Parm1	x	x		
Bcat1	x	x		
Dnm3	x	x		
Nt5e	x	x		
Rassf6	x	x		
Tox	x	x		
Ifit8	x		x	
Ifit2	x		x	
Slfn5	x		x	
Rsad2	x		x	
Lyg6c2	x		x	
Pydc3	x		x	
Ighv1-69	x		x	
Ifi44	x		x	
Oas1g	x		x	
Slfn1	x		x	
Gm14446	x		x	
Ifit1	x		x	
Mx1	x		x	
Irf7	x		x	
Oas3	x		x	
Usp18	x		x	
Oas1a	x		x	
Ly6a	x		x	
Rtp4	x		x	
Ifi27I2a	x		x	
Usp40		x	x	x
Tfe3		x		x
Pdgfc		x		
B				
Downregulated Genes (q<0.05 & Log2<1)	Mitf^mi-vga9/mi-vga9^	TDN-B	Mitf^mi-vga9/+^	TDN-BT
Gm18752		x		
Oas1g		x		
Pdgfc	x			X
Usp40	x			
Pkd1l1	x	x	x	
Usp40	x			
Nnt	x		x	
Camk2b	x		x	
9030025P20Rik	x		x	
Cpne4	x		x	

Upregulated (A) and Downregulated (B) genes based on likelihood ratio q<0.05 and Log2 fold after change > |1|, represented as x, in each Mitf/MiT family impairment mouse model (TDN-B, TDN-BT, Mitf^mi-vga9/mi-vga9^, and Mitf^mi-vga9/mi-vga9^) B cells relative to wildtype B cells.

**Figure 6:**
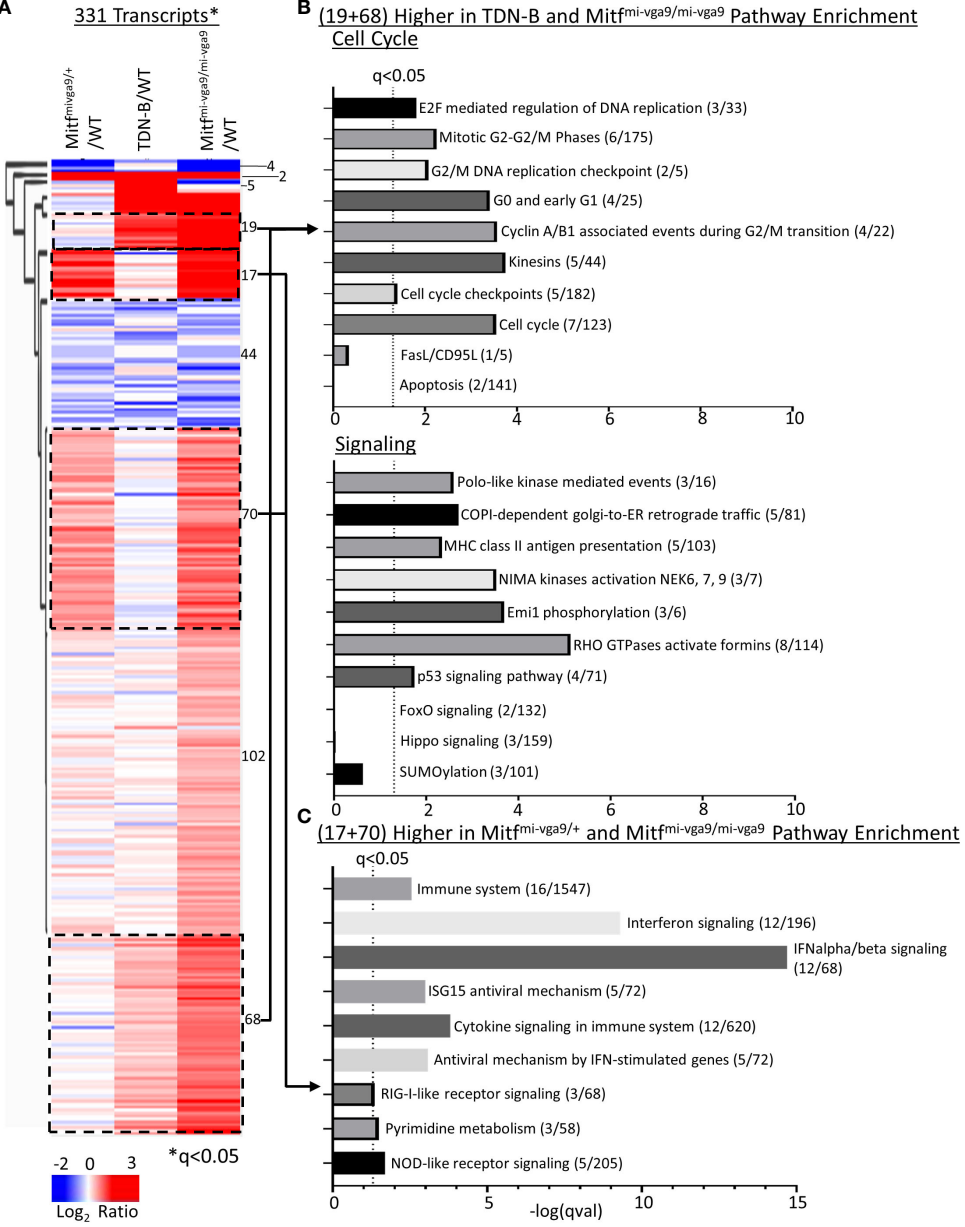
Pathway enrichment identified through clustering of Mitf^mi-vga9/+^, TDN-B, and Mitf^mi-vga9/mi-vga9^ transcript expression. **(A)** Unsupervised hierarchical clustering of significantly differentially expressed transcripts. Encircled clusters represent clusters used in pathway enrichment analysis. Clustering with average Euclidean distance linkage. Red is high, blue is low. Likelihood ratio q<0.05. **(B)** Pathway enrichment analysis of transcripts with increased expression in both TDN-B and Mitf^mi-vga9/mi-vga9^ B cells. **(C)** Pathway enrichment analysis of transcripts with increased expression in both Mitf^mi-vga9/+^ and Mitf*^mi-vga9/mi-vga9^* B cells. Pathway analysis performed with Enrichr, using Reactome and KEGG annotations. Dashed line indicates Fisher’s exact test qval<0.05. X axis indicates the –log(qVal) for each pathway.

To systematically relate biological processes to corresponding representative transcripts that might be enriched among those differentially expressed, we applied hierarchical clustering analysis across all models (WT, TDN-B, TDN-B/T, Mitf^mi-vga9/+^, and Mitf^mi-vga9/mi-vga9^) with the 331 transcripts significantly differentially expressed (likelihood q<0.05) (**Figure 6A**). Subsequent pathway enrichment analysis of clusters was completed using Enrichr (11) of clusters with increased expression (**Figures 6A–C**). Of the cluster with 44 transcripts with decreased expression in TDN-B, TDN-B/T, and Mitf^mi-vga9/mi-vga9^ models compared to wild type, there were no significant pathway enrichments by Fisher’s exact test (q<0.05) based on Reactome (29) pathway analysis. In concordance with evidence for increased B cell activation and GCs in Mitf^mi-vga9/mi-vga9^ and TDN-B mice, pathways relevant to activated B cell proliferation, including cell cycle, DNA replication, phosphorylation, kinase activation, and antigen presentation, were enriched in two clusters with 19 and 68 genes in each, with higher expression in TDN-B and Mitf^mi-vga9/mi-vga9^ B cells compared to controls (**Figure 6B**). Consistent with elevated Ly6A/E expression on Mitf^mi-vga9/mi-vga9^ B cells detected by flow cytometry, Ly6a transcript expression was also significantly increased by RNAseq in Mitf^mi-vga9/mi-vga9^ B cells (**Supplementary Table 1**). Further, pathways relevant to interferon signaling, cytokine signaling, and metabolism were enriched in two clusters of 17 and 70 genes each, with higher expression in Mitf^mi-vga9/+^ and Mitf^mi-vga9/mi-vga9^ B cells compared to TDN-B and wild-type controls (**Figure 6C**). In contrast, pathways including apoptosis known to be regulated by Mitf and MiT family members in other cell types were not significantly enriched in these Mitf^mi-vga9/+^ and Mitf^mi-vga9/mi-vga9^ B cell RNAseq clusters. Consistent with these findings, significant differences in apoptosis of naïve B cells in culture were not observed through flow cytometric and LPS or IgM + CD40 stimulation functional assays (**Supplementary Figure 7**).

**Figure 7:**
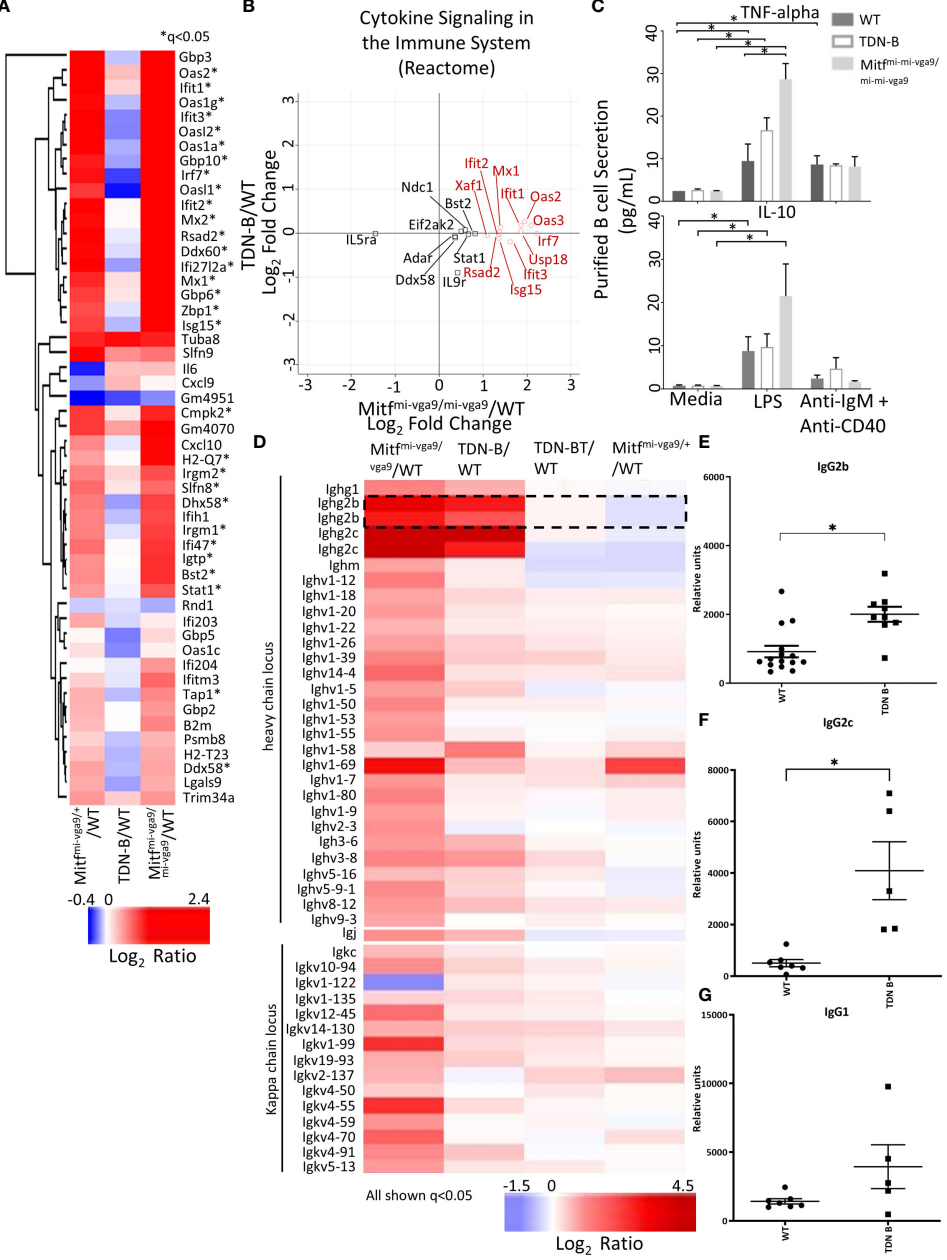
Differential expression of transcripts related to cytokine secretion and immunoglobulins with Mitf and/or MiT/TFE family functional impairment. **(A)** Heatmap visualization of transcripts indicated in Harris et al., 2018 to participate in innate immune signaling and type I interferon responses. *q<0.05 likelihood ratio in the present dataset. **(B)** Scatterplot visualization of transcripts differentially expressed within the Reactome pathway, “cytokine signaling in the immune system.” Red circles indicate genes that have >+1 in Mitf^mi-vga9/mi-vga9^ compared to wildtype B cells. 3 months old, female mouse purified B cells. **(C)** Concentration of TNF-alpha and IL-10 in the supernatant of purified B cell cultures after LPS and anti-IgM + anti-CD40 stimulation. Age and sex matched, male and female 3-4 months old mouse purified B cells. Kruskal-wallis w/multiple comparisons *post-hoc*, error bars indicate SEM, *p<0.05. **(D)** Heatmap of significantly differentially expressed transcripts from the heavy chain locus, light chain locus, and joining chain. IgG2b transcripts are encircled. 3 months old, female mouse purified B cells. **(E–G)** TNP specific IgG2c, IgG2b, and IgG1 sera levels seven days after immunization in wildtype and TDN-B mice with TNP-Ficoll. Age and sex matched, male and female 8-10 months old mice. Kruskal-wallis w/multiple comparisons *post-hoc*, error bars indicate SEM, *p <0.05.

Using RNAseq, Harris et al. (2018) (19) reported the upregulation of 55 genes of innate immune signaling in Mitf^mi-vga9/+^ melanocyte stem cells, supporting a role of Mitf in innate immune response. Among those 55 genes, we found that 30 genes were similarly increased in Mitf^mi-vga9/+^ and Mitf^mi-vga9/mi-vga9^ B cells (**Figure 7A**, marked by asterisk*). However, these changes were not observed in TDN-B B cells (**Figure 7B**), a finding consistent with the flow cytometry results for Ly6 expression (**Figure 1F**). Similarly, multiplex immunoassays showed elevated expression of TNF-alpha in Mitf^mi-vga9/mi-vga9^ B cell culture supernatants after LPS stimulation, but not in TDN-B B cell supernatants (**Figure 7C**). No differences were seen in the production of IL-10 between groups with either LPS or anti-IgM+anti-CD40 stimulation. The RNAseq data indicated that in both TDN-B and Mitf^mi-vga9/mi-vga9^ B cells, there was increased expression of immunoglobulin genes from the heavy chain and kappa chain locus, especially IgG2b and IgG2c (**Figure 7D**). Consistent with this finding, serum titers of TNP-specific IgG2b and IgG2c, but not IgG1, were significantly increased in the sera from TDN-B mice seven days after immunization with TNP-ficoll when compared to sera from wild type mice (**Figures 7E–G**).

### 3.6 Comparison of TDN-B and TDN-B/T B cell transcripts helps delineate T helper cell dependent pathways

**Figure 8:**
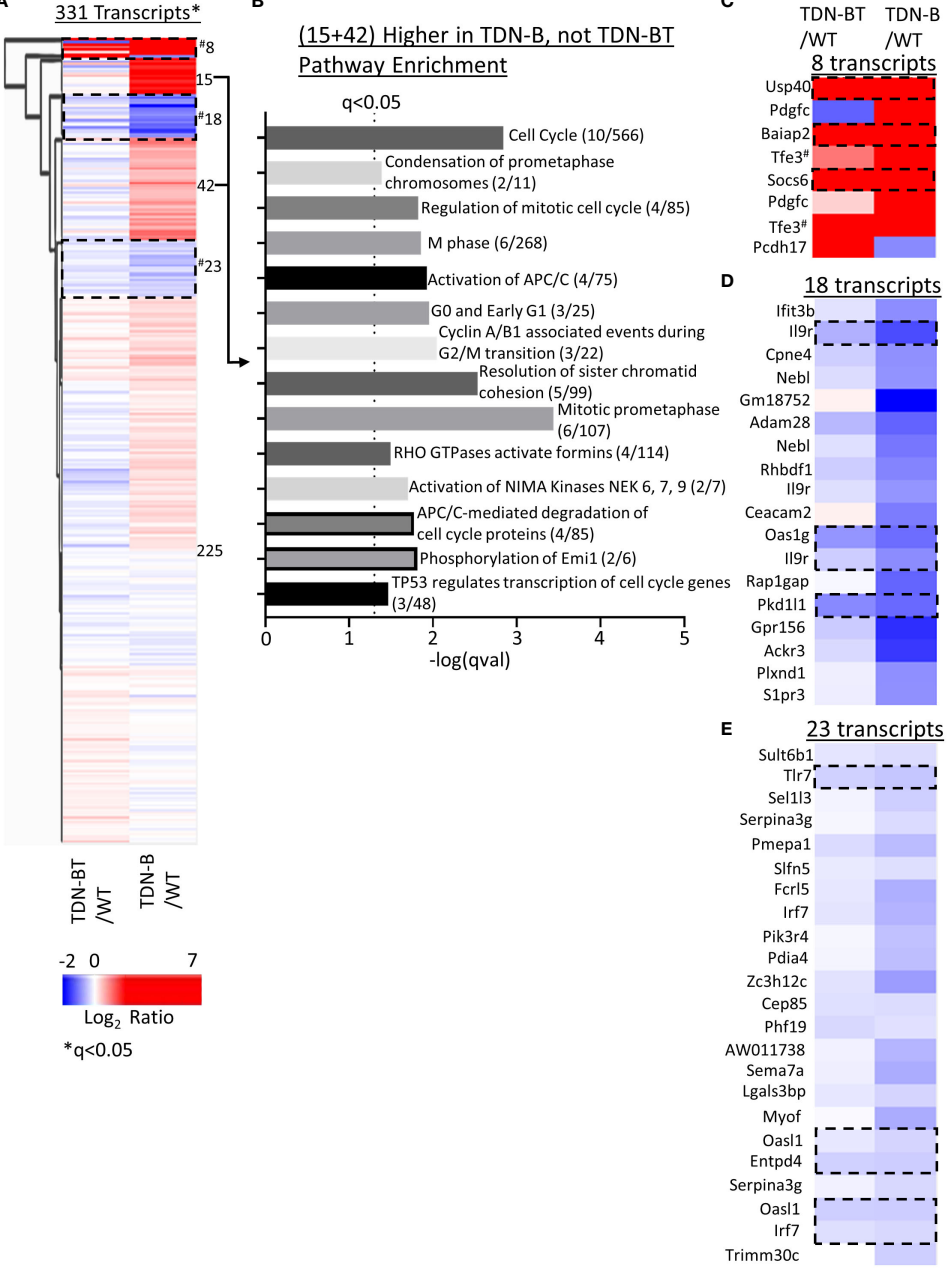
Pathway enrichment identified through clustering of TDN-B and TDN-BT transcript expression. **(A)** Unsupervised hierarchical clustering of significantly differentially expressed transcripts. Encircled clusters represent clusters used in pathway enrichment analysis. Clustering with average Euclidean distance linkage. Red is high, blue is low. Likelihood ratio q<0.05. **(B)** Pathway enrichment analysis of transcripts with increased expression in only TDN-B, but not TDN-BT B cells. **(C–E)** Identification of select clusters from (A). Encircled transcripts represent transcripts increased or decreased in both TDN-B and TDN-BT B cells. Pathway analysis performed with Enrichr, using Reactome and KEGG annotations. Dashed line indicates Fisher’s exact test qval<0.05. ^#^indicates two tfe3 transcripts that are increased in both TDN-B and BT due to their identity to the transdominant negative transgene.

We next sought to compare gene expression patterns in TDN-B and TDN-B/T B cell through heat map visualization to assess the B cell transcripts influenced by T cell help, which is impaired due to TDN expression in T cells in the TDN-B/T line. While most transcripts showed minimal differential expression compared to wild type (**Figure 8A**, Cluster 225), two clusters enriched with cell cycle, cell division, and kinase activation signaling pathways showed increased transcripts in TDN-B, but not TDN-B/T B cells (**Figure 8A**, Clusters 15 and 42), suggesting an effect of T cell help on MiT function in B cells (**Figure 8B**). The T cell independent MiT activities were revealed by the finding of commonly changed transcripts in TDN-B and TDN-B/T B cells as compared to wild type (Clusters 8,18,23, **Figures 8A, C–E**). These transcripts include highly upregulated Usp40, Baiap2, and Socs6 (Cluster 8) and downregulated Il9r, Pkd1l1, Tlr7, Oasl1, Entpd4, and Irf7 (Cluster 18 and 23). Notably, Tfe3 was expected to be higher in both models than in WT because the TDN gene was constructed from *Tfe3* cDNA sequences, and which served to independently validate the RNAseq. **Supplementary Figure 8** summarizes gene expression changes observed in TDN-B and Mitf^mi-vga9/mi-vga9^ B cells.

### 3.7 Defining differentially expressed transcripts in TDN-B and Mitf^mi-vga9/mi-vga9^ B cells

**Figure 9:**
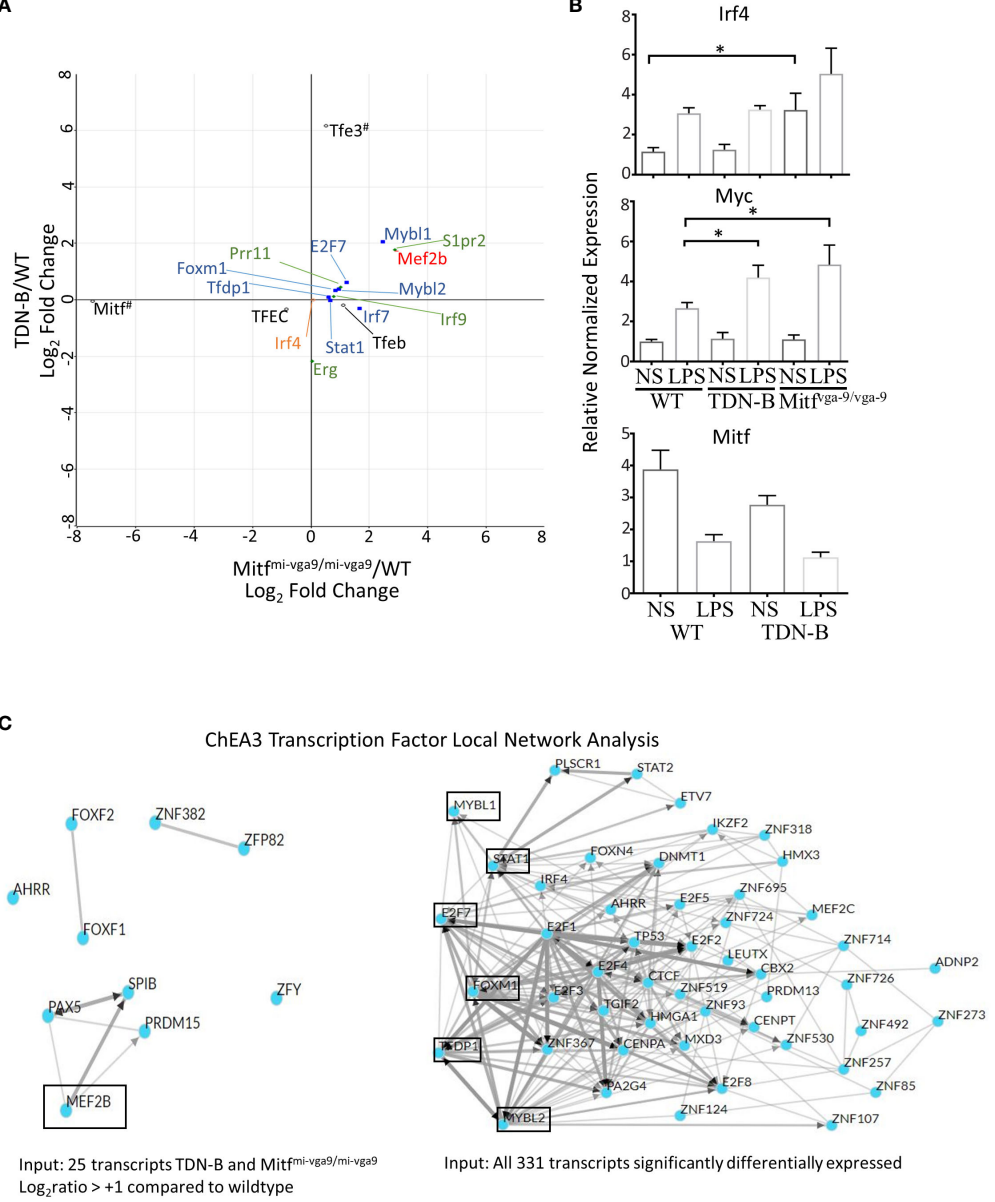
Prediction analysis and consideration of theoretical direct binding sites of Mitf and/or MiT family members in B cells. **(A)** Scatterplot of select transcripts mapped a the log2ratio of TDN-B/WT against Mitf^mi-vga9/mi-vga9^/WT. MiT/TFE family members are indicated in black, open circles. Blue squares and red circle are only transcription factors from **(C)**. Green diamonds indicate Mitf direct gene binding targets in mast cells, as observed through ChiP-Seq in Calero-Nieto, et al., 2014. All transcripts depicted are significantly differentially expressed, except Irf4, which is indicated by an orange circle. ^#^Indicates that Mitf has low Mitf^mi-vga9/mi-vga9^/WT relative expression, because Mitf has minimal to no expression in Mitf^mi-vga9/mi-vga9^ mice and that Tfe3 is indicated to have high TDN-B/WT relative expression because the transdominant negative transgene (TDN) is likely recognized as a Tfe3 transcript during RNAseq analysis. **(B)** Relative normalized expression of select genes, Irf4, Myc, and Mitf, with and without LPS stimulation in purified, naïve B cells. Data shown, age and gender matched male and female mouse purified B cells. 2-4 months old. **(C)** ChIP-X enrichment analysis 3 (ChEA3) prediction analysis of the transcript factor network related to transcripts significantly differentially expressed in B cells with Mitf and/or MiT/TFE family functional impairment. (Left) Local network visualization of 10 top-ranked transcription factors identified through ChEA3 based on the 25 transcripts in **Table 1**. Mef2b is encircled because it is significantly increased in TDN-B and Mitf^mi-vga9/mi-vga9^ B cells. (Right) Local network visualization of 50 top-ranked transcription factors identified through ChEA3 based on all 311 significantly differentially expressed transcripts in the present study. Encircled transcripts are transcription factors also identified as significantly differentially expressed in the present study. (Mybl1, Stat1, E2f7, Foxm1, Tfdp1, Mybl2). Additionally, two other transcription factors, Mef2b and Irf7, were identified as Top-ranked based on this gene set input by ChEA3 at ranks 58 and 81, respectively.

As shown in **Figure 9A**, an unexpected finding was that Irf4 transcript abundance was no different than wild type in Mitf^mi-vga9/mi-vga9^ or TDN-B B cells, residing at almost 0;0 in each comparison (**Figure 9A**, orange). Consistent with that finding, RT-qPCR results showed no statistically significant difference in Irf4 mRNA between control and TDN-B cells, whereas there was only a 2-3-fold increase of Irf4 mRNA in unstimulated Mitf^mi-vga9/mi-vga9^ B cells compared to WT or TDN-B B cells (**Figure 9B**). In contrast, LPS stimulation increased Irf4 expression about 2-fold in WT and TDN-B B cells, but not in Mitf^mi-vga9/mi-vga9^ B cells. This is in contrast to the dramatic increase of basal (> 4 fold) and inducible (> 5 fold) Irf4 expression in Mitf^mi/mi^ B cells reported by Lin et al. (1). We believe that this difference possibly reflects the effects of MiT inactivation in white blood cells other than B cells in the Mitf^mi/mi^ HSCT model, which is supported by Lin et al.’s conclusion that the binding site for Mitf on Irf4 promoters could not be found (1). Interestingly, genes that have been found in prior studies to be directly bound by Mitf, such as Prr11, S1pr2, Erg, and Irf9, which were identified by a ChIP-Seq analysis of mouse mast cells (30), were differentially expressed in TDN-B and Mitf^mi-vga9/mi-vga9^ B cells (**Figure 9A**, green). In contrast to Irf4, the abundance of Myc transcripts was greater in TDN-B and Mitf^mi-vga9/mi-vga9^ B cells compared to wild type after 24 hours in culture with LPS stimulation (**Figure 9B**), consistent with the hypothesis that mitogenic stimulation may drive increased proliferation in B cells that have impaired Mitf function, as compared to wild type B cells.

Given that transcription factors were among those differentially expressed in Mitf/MiT-deficient B cells, we sought to predict what transcription factors other than Mitf/MiT might participate in regulation of the differentially expressed transcripts. We performed a transcription factor enrichment analysis using ChIP-x enrichment analysis 3 (ChEA3) (12), a gene-set enrichment analysis tool tailored to test if query gene-sets are enriched with genes that are known putative targets of transcription factors. As shown in **Figure 9C**, one of the 10 Top-ranked transcription factors predicted by ChEA3 analysis was Mef2b, which was found significantly increased in TDN-B and Mitf^mi-vga9/mi-vga9^ B cells compared to wild type. Therefore, Mef2b is a possible downstream target that is normally repressed by Mitf in B cells (**Figure 9C**). In addition to Mef2b, Mybl1, Mybl2, Foxm1, E2f7, Tfdp1, and Stat1 were also predicated by ChEA3 within the 50 Top-ranked transcription factors associated with the 331 transcripts significantly differentially expressed, all of which were also significantly differentially expressed in TDN-B and Mitf^mi-vga9/mi-vga9^ B cells compared to wild type (**Figure 9A**, Blue). **Supplementary Figure 8** provides a summary Venn diagram of phenotypic and select transcription abnormalities observed in TDN-B vs. Mitf^mi-vga9/mi-vga9^ mice.

### 3.8 Genes differentially expressed with Mitf and MiT family impairment are also differentially expressed in normal human B cell subsets

We next sought to consider how B cell gene expression patterns observed in our Mitf and MiT family deficient mouse models relate to human B cell biology. Using SAM (Significance Analysis of Microarray) multiclass analysis, Kassambara et al. (2015) (13) defined 9303 genes significantly differentially expressed in different subsets of human B cells including naïve B cells, memory B cells, centroblasts, centrocytes, preplasmablasts, plasmablasts, early plasma cells, and bone marrow mature plasma cells.

**Figure 10:**
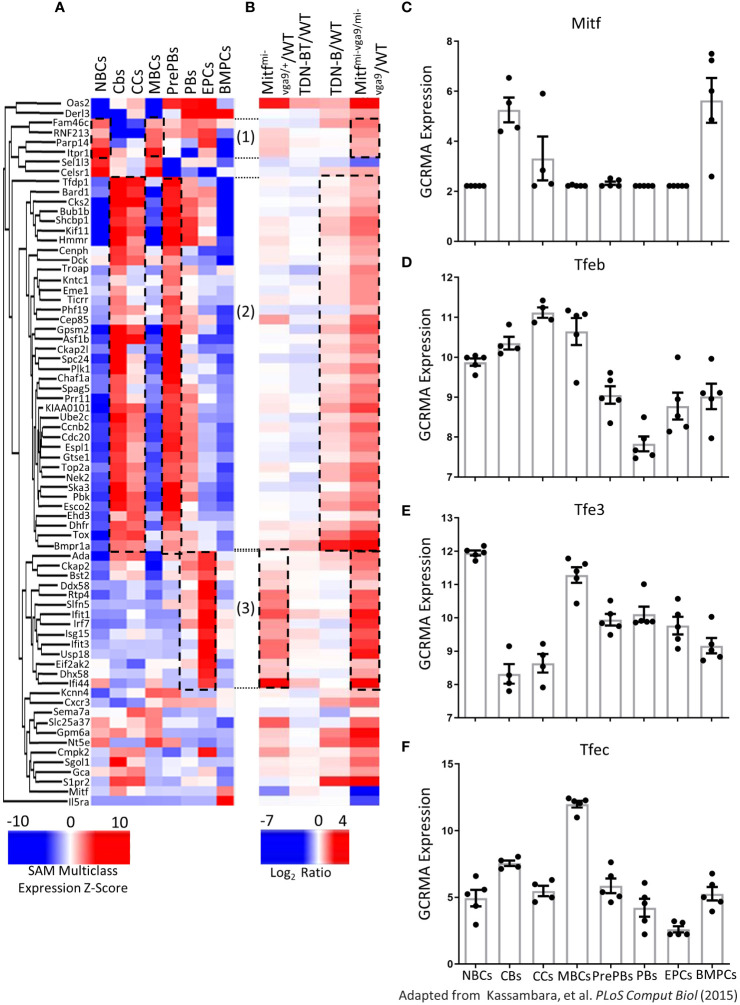
Expression of MiT family members in human B cells and plasma cells. **(A, B)** Heatmap visualizing 72 transcripts significantly differentially expressed in B cells after Mitf and/or MiT/TFE family functional impairment also identified by Kassambara et al., 2015 (13) to be differentially expressed in normal B cell development stages. **(A)** Unsupervised hierarchical clustering of 72 transcripts quantified based on significance analysis of microarrays multiclass expression z-scores access through genomicscape. **(B)** 72 transcripts, aligned with transcripts in **(A)** quantified as log2ratio of B cell transcripts in each Mitf and MiT/TFE functional impairment model relative to wildtype. Encircled transcripts indicate those with a similar pattern of expression in Mitf^mi-vga9/mi-vga9^ naïve B cells and human B cell subsets. 3 months old, female mouse purified B cells. **(C)** Mitf **(D)** Tfeb **(E)** Tfe3, and **(F)** Tfec Log2 GC robust multi-array average quantification identified as significantly differentially expressed across eight mature B cell stages and plasma cells. Adapted from Kassambara et al., 2015 (13). Naïve B cells (NBCs), germinal center centroblasts (CBs), germinal center centrocytes (CCs), memory B cells (MBCs), preplasmablasts (PrePBs), early plasma cells (EPCs), plasmablasts (PBs), and bone marrow plasma cells (BMPCs).

Interestingly, by comparing the expression data from Kassambara et al., 2015 (13) to our mouse gene datasets, we observed that 72 genes were significantly differentially expressed in both normal human B cell subsets and mouse B cells with Mitf and MiT family impairment (**Figures 10A, B**). Specifically, transcripts increased in human naïve B cells and memory B cells were also increased in Mitf^mi-vga9/mi-vga9^ mouse B cells; transcripts that were increased in human centroblasts, centrocytes, and pre-plasmablasts were also increased in Mitf^mi-vga9/mi-vga9^ and TDN-B mouse B cells, and transcripts that were increased in human plasmablasts and early plasma cells were also increased in Mitf^mi-vga9/+^ and Mitf^mi-vga9/mi-vga9^ mouse B cells (**Figures 10A, B**, dotted borders).

Distribution of expression of MiT family members across B and plasma cell developmental stages are also shown (**Figures 10C–F**). All four MiT family members were significantly differentially expressed among these clusters (**Supplementary Table 3**). For instance, among the MIT family members, TFE3 and TFEB were expressed in resting mature B cells, while Mitf and TFEC were not. In contrast, TFEC expression was highest in memory B cells. TFE3 and TFEB were also present in memory B cells, whereas Mitf was not detected in this subset (**Figure 10**). **Supplementary Figure 9** shares the distribution of expression of other significantly differentially expressed genes in MiT mouse model B cells (Mybl1, Mybl2, Mef2b, Foxm1, Stat1, E2f7, and Socs6), as well as Irf4. Overall, this meta-analysis shows that genes differentially regulated in naïve B cells from our Mitf- and MiT-deficient mouse models have similar expression patterns corresponding to specific developmental stages and mature subsets of human B cells, supporting a regulatory role of Mitf and MiT in B cells and the importance of Mitf target genes in human B cell development.

## 4 Discussion

Although the study of Mitf in B cells started over three decades ago (1, 31), how Mitf exerts its function through downstream target genes and pathway modulation in B cells has remained obscure. Our study emphasizes and clarifies the importance of Mitf to B cell homeostasis, activation, and tolerance, using distinct, but related Mitf-deficient mouse models, which complement the original HSCT model (1) that first identified this key relationship. These models also provide more detailed evidence of the range of abnormalities in lymphocyte compartments caused by lack of Mitf function, either in B cells or systemically, and provide new data about the gene expression program Mitf influences to restrain B cell activation and entry into the germinal cell/plasma cell (GC/PC) developmental program. Unexpectedly, these findings also challenge a long-standing model that proposed that Mitf restrains GC and PC development through repressing Irf4 expression. In sum, the data presented here, through complementary models of Mitf functional impairment, are consistent with a model in which Mitf regulates genes in a B cell intrinsic manner that is further influenced through T helper cell dependent process, in which dysregulation caused by Mitf inactivation drives B cell subset abnormalities and causes splenomegaly, elevated auto-antibodies, and proteinuria.

### 4.1 Immune abnormalities in different models for Mitf/MiT inactivation point to a pivotal role of Mitf

Comparing findings among the different models for Mitf/MiT inactivation provided a systematic approach to resolve differences between the effects of systemic versus B cell intrinsic functions. Also, these models begin to differentiate null versus trans-dominant effects, the latter of which may influence activities of other MiT family members and aspects of cell metabolism they might uniquely control. From this vantage point, it is noteworthy that all three models - TDN-B, Mitf^mi-vga9/mi-vga9^, and the Mitf*^mi/mi^* HSCT chimera - have an increased spontaneous frequency of plasma cells, produce elevated serum immunoglobulins, and elevated autoantibodies, including rheumatoid factor and anti-DNA. This supports the validity of using all three models to explore B cell effects of Mitf and MiT family functional inactivation and dissect which genes are influenced under which set of circumstances.

Other related, but previously undocumented spontaneous abnormalities in TDN-B and Mitf^mi-vga9/mi-vga9^ mice not described in the HSCT model (1), include splenomegaly, mild proteinuria, and changes to splenic lymphoid architecture. Changes to lymphocyte populations included elevated PCs/GCs in bone marrow and lymph nodes, increased frequency of Ly6A/E+Ly6C+ B cells, and changes in the frequencies of CCR7^+^ and CD62L^lo^CD44^hi^ CD4 T-cell subsets. Additionally, transcription upregulation of genes linked to germinal center regulation, plasma cell differentiation, and B cell activation were upregulated in TDN-B and Mitf^mi-vga9/mi-vga9^ mouse B cells, including Mef2b (32), Aicda (24), Rgs13 (25), S1pr2 (26), Mef2b (27), Nuggc (28), IgG2b, and IgG2c, among others (**Table 1**). The upregulation of these genes and the phenotypic changes observed suggest there is a breach in B cell tolerance in TDN-B and Mitf^mi-vga9/mi-vga9^ mice whereby Mitf and MiT family members’ loss-of-function permits increased transcriptional expression of genes that promote increased B cell activation and dysregulation of the germinal center/plasma cell developmental program. Although many of these phenotypes were not described in the adoptive transfer HSCT system (1), the present study evaluated mouse B cells at 3-8 months of age, a time period which may have allowed the emergence of phenotypes that may require particular developmental contexts and/or environmental exposures to evolve.

Comparisons of the TDN-B and Mitf^mi-vga9/mi-vga9^ models to TDN-B/T mice suggested that many, but not all, B cell abnormalities were T-helper cell dependent. The discrete B cell – T cell partition observed in splenic follicles of un-immunized wild type mice was disrupted in TDN-B and Mitf^mi-vga9/mi-vga9^ mice, but remained intact in TDN-B/T mice, suggesting that B cell intrinsic processes that control partitioning are regulated by Mitf. Although disorganization of follicles correlated with the elevated frequency of GC and plasma cells, further studies are required to define the relationship between these phenomena and the underlying mechanisms responsible.

Importantly, these phenomena appear independent of a generalized elevated type I IFN signature caused by systemic Mitf deficiency that was evidenced by expression of Ly6 on B lymphocytes from Mitf^mi-vga9/mi-vga9^, but not TDN-B mice. Previously, an altered microenvironment, proposed to be due to increased production of type 1 interferons, was suggested by the effects observed on melanocyte progenitor stem cells in Mitf^mi-vga9/+^ mice (19). Although both Mitf^mi-vga9/mi-vga9^ mice and TDN-B mice produced anti-DNA antibodies, this elevated type 1 interferon gene signature might help explain why anti-dsDNA antibody was produced in Mitf^mi-vga9/mi-vga9^ mice, whereas anti-ssDNA was made in TDN-B mice. Greater evidence of dysregulated B cell activation *in vitro* was also observed in Mitf^mi-vga9/mi-vga9^ mice, specifically, enhanced secretion of TNF-alpha *in vitro* by Mitf^mi-vga9/mi-vga9^ B cells and increased c-myc expression in TDN-B B cells after LPS stimulation. However, further work is needed to determine whether the enhanced responsiveness of B cells from Mitf^mi-vga9/mi-vga9^ mice to mitogenic stimuli was intrinsic to Mitf deficiency itself, or due to cell interactions such as T-cell dependent signaling or development in an Mitf-deficient cytokine milieu that persisted in culture. Further work is also required to determine if similar increased proliferative signatures are present in Mitf-inactivated B cells obtained from microenvironments that lack T cell help.

### 4.2 MITF-mediated regulation of B cell responses

Our findings further establish Mitf as a key player in regulating B cell responses through restraining B cell activation and PC development. Interestingly, Mitf seems to act as a major repressor of gene expression in B-cells. Unexpectedly, our RNAseq analysis showed that Irf4 expression was unchanged in ex-vivo B cells from all four models of Mitf inactivation that we used (Mitf^mi-vga9/mi-vga9^, TDN-B, TDN-B/T, Mitf^mi-vga9/+^), a finding largely confirmed in cultured TDN-B and Mitf^mi-vga9/mi-vga9^ B cells via RT-PCR. After 20-24 hour culture, basal and LPS-induced levels of Irf4 were modestly (~2-fold) higher than wild-type in Mitf^mi-vga9/mi-vga9^ B cells, whereas Irf4 levels in TDN-B B cells were comparable to wild type. This is in contrast to the Mitf*^mi^* HSCT model (1) in which orders of magnitude increases in Irf4 expression were observed in Mitf deficient B cells. Our findings do not provide evidence to support a role for Mitf as a repressor of Irf4 in B cells. Although it is not clear yet what the underlying reasons are for these differences in Irf4 expression in different model systems, given that in the Mitf*^mi^* HSCT model the entire MiT family is also potentially inactivated in hematopoietic cells other than B cells, upregulation of Irf4 transcription in the Mitf*^mi^* HSCT model by non-B cell factors cannot be excluded. Interestingly, in melanocytes Mitf is a direct activator of Irf4, indicating cell type specificity in how this transcription factor network is regulated (33).

Transcription factor enrichment analysis (12) demonstrated that Mef2b is a Top-ranked transcription factor that is predicted to be part of the transcription factor network responsible for regulating gene expression in both TDN-B and Mitf^mi-vga9/mi-vga9^ naïve B cells. Since Mef2b is a known transcription factor that regulates B cell differentiation to germinal center B cells (34) and is included in a network with other transcription factors known to regulate B cell to plasma cell differentiation (**Figure 9C**), the observed transcriptional regulation of Mef2b by Mitf in B cells could define an alternate pathway to that proposed by Lin et al., 2004 linking Mitf from B cell to plasma cell development. Additional alternate pathways are supported by Top-ranked transcription factor network analysis of the 311 genes with significant differential expression in B cells across Mitf and MiT family impairment models. Overall, differential transcription factor expression in TDN-B and Mitf^mi-vga9/mi-vga9^ B cells supports the existence of a network of gene transcription that is B cell autonomous, correlating with B cell activation and regulation. Select transcription factors known to directly regulate B cell to plasma cell differentiation, including Irf4, E2f1, and Mef2c, were not significantly differentially expressed in TDN-B and Mitf^mi-vga9/mi-vga9^ B cells, but were included in transcription factor network mapping, supporting indirect, perhaps non-autonomous B cell roles affected by Mitf and MiT family members.

Changes in the Mef2b network are consistent with evidence of germinal center program dysregulation given that the molecules in this network have known roles in B cell differentiation and germinal center processes. Particularly, Aicda (AID) and Nuggc (also known as SLIP-GC) have been shown to have similar expression patterns (28) in germinal center B cells: Aicda is necessary for somatic hypermutation (SHM) and class switch recombination (35); and increased Aicda expression in TDN-B and Mitf^mi-vga9/mi-vga9^ supports that Mitf is a negative regulator of Aicda. Further studies are required to determine whether Aicda has promoters directly regulated by Mitf.

Another gene repressed by Mitf, Rgs13, is known to regulate cell cycle and germinal center specific genes. Rgs13 regulates extra-follicular plasma cell generation, germinal center size, and germinal center B cell numbers (25). The finding that the expression of Rgs13 is increased upon Mitf loss-of-function indicates perturbations in naïve B cell homeostasis and may help explain the spontaneous disorganization of many follicles observed in TDN-B and Mitf^mi-vga9/mi-vga9^ mice. Interestingly, Rgs13 (36), and another differentially expressed gene, S1pr2 (26), are both involved in germinal center confinement of B cells. S1pr2, through binding with S1P, defines a gradient that restricts B cell movement within the germinal center. Further studies are needed to discern whether and how perturbation of Rgs13 and S1pr2 expression through Mitf inactivation could explain the observed changes in B and T cell localization.

Socs6 and Baiap2 emerge as two new novel targets of Mitf-mediated repression. The expression of both genes is dramatically increased across all four models of Mitf inactivation (Mitf^mi-vga9/mi-vga9^, TDN-B, TDN-B/T, and Mitf^mi-vga9/+^), but their specific mechanisms of involvement in B cell regulation requires further investigation. Socs6 regulates cytokine signaling, including Kit receptor functions (37), and given that Kit regulates lymphocyte development (38), it may contribute to the lymphocyte disorganization and/or disrupted B cell homeostasis observed in this study. Baiap2, also known as IRSp53, is a scaffold/adaptor protein that regulates membrane dynamics with Cdc42 (39), which may affect B cell activation and differentiation (40). However, given that splenomegaly, RF, and elevated urine protein were not observed in all lines, the increased expression of these genes is either not relevant or not sufficient to cause these B cell abnormalities. Regardless, the changes driven by Socs6 and Baiap2 warrant further study.

Interestingly, a small number of transcripts, including Usp40, Pkd1l1, Pdgfc, and Cpne4, have significantly decreased relative expression (lrt q<0.05, FC < -1) in Mitf^mi-vga9/mi-vga9^ B cells compared to wild type. Whether the decreased expression of these transcripts, particularly Pdgfc and Usp40, contributes to primary follicle disorganization in Mitf^mi-vga9/mi-vga9^ mice remains to be determined. Given that these changes are unique to the Mitf-null model, the relationship of these genes to Mitf function in B cells and the influence of their interactions with other MiT members and other cell types remain unclear. One consideration includes further study into the downstream effects on apoptosis that may result from decreased expression of Usp40 and Pdgfc in Mitf-null model B cells. Further study into Usp40 and Pdgfc is especially intriguing to consider because it is also unclear why Pdgfc is increased in TDN-B only, but low in all other models. Pdgfc has been reported to enhance Erk activation and adhesion-independent growth of pre-B cell lines (41), but its role in naïve and activated B cells is still unknown. Whether or not the increased Pdgfc in TDN-B B cells, but not Mitf^mi-vga9/mi-vga9^ B cells, is linked to increased pre-B cell proportions in TDN-B mice only (**Supplementary Figure 3**) requires further investigation. Usp40, on the other hand, was increased in TDN-B, TDN-B/T, and Mitf^mi-vga9/+^, but was absent from Mitf^mi-vga9/mi-vga9^ B cells. We theorize that Usp40 may be influenced by Mitf gene dosage and may also be impacted by the complete loss-of-function of Mitf in non-B cells. Further work is needed to determine the importance of these findings to B cell biology and how they are regulated by the members of the MiT family.

In other cell types, Tfeb and Mitf have been defined as master regulators of autophagy (42, 43) and Mitf has been shown to drive endolysosomal biogenesis (44). However, our study did not discover any changes in the expression patterns of genes related to autophagy, mitophagy, and lysosomal biogenesis. These include mTOR, Atg5, Atg7, all V-type proton ATPase subunits, RagA,B,C, and D, and LAMP1 and 3 (**Supplementary Table 1**). Mitf has also been shown to regulate genes involved in reactive oxygen species, lysosome biogenesis/autophagy important for antigen presentation, PC development, and B cell activation/GC responses (42), as have Tfe3 and Tfeb (45). An important direction for future studies will be to analyze how Mitf-dependent pathways interact with these processes.

No changes were observed in the expression of CD84 or Ly108 (Slamf6), both of which are implicated in restraining GC development and preventing the production of lupus autoantibodies (46). On the other hand, T-Bet (Tbx21), which is associated with age-related ABC B cells, was significantly differentially expressed in Mitf^mi-vga9/mi-vga9^ B cells compared to wild type and may be a downstream regulator of spontaneous germinal centers that are a source of autoantibodies (47). However, it does not seem to be responsible for the B cell abnormalities shared by TDN-B and Mitf^mi-vga9/mi-vga9^ mice since it was not upregulated in TDN-B B cells. In addition, our data indicate that pathways related to apoptosis (48), FoxO signaling (49), and SUMOlyation (50) were not significantly enriched in our pathway analysis of TDN-B and Mitf^mi-vga9/mi-vga9^ B cells.

Lastly, prior published data in human cells, including naïve B cells, germinal center B cells, memory B cells, and plasma cells, demonstrated significant differential expression of MiT family members (**Figure 10**). Further, many genes differentially expressed among normal human B cell subsets corresponded to those found to be regulated by Mitf in mouse B cells in the present study, justifying future work in human B cells to explore how Mitf and MiT family members contribute to human B cell development and B cell-driven disease. It will also be important to further analyze the B-cell receptor repertoire in TDN-B and Mitf^mi-vga9/mi-vga9^ B cell and plasma cell populations to explore how that may relate to their abnormal expansion and pathological antibody specificities, and compare those to the B-cell repertoires of SLE patient samples. Given that human memory B cells showed differential expression of MiT family member genes (**Figure 10**), and since B cell subsets that may differentiate into germinal center-dependent or germinal center-independent memory B cells (CD19+CD38+GL7+) were significantly increased in Mitf^mi-vga9/mi-vga9^ mice (**Figure 1D**), these data provide direction for future functional studies that focus on the role of MiT members in memory B cell differentiation.

### 4.3 Mitf and autoimmunity

Despite the autoimmune features exhibited by TDN-B and Mitf-null mice, the enhancement of certain autoimmune-associated abnormalities in the TDNB.Fas^lpr/lpr^ mice, and the “interferon signature” of systemic Mitf insufficiency (51), how Mitf relates to lupus and whether Mitf has an involvement in human autoimmune disease remain unclear. Lupus-like symptoms have not been reported in individuals that lack Mitf gene function. The interaction of MiT inactivation and the lpr mutation was a starting point to identify in what possible capacities Mitf/MiT might have such a role and through which pathways, which in turn could be influenced by known autoimmune mechanisms. That anti-chromatin titers were not affected, and that anti-DNA titers were elevated, seem to indicate some selectivity to self-reactivity influenced by Mitf. It should be noted that there were no discernable perturbations in the expression patterns of genes involved in mediating TLR pathways or other genes known to be dysregulated in other mouse lupus models. However, given that autoimmune symptoms develop with B cell specific loss of function of MiT family members, this warrants further exploration of the relationship of Mitf to B cell tolerance.

### 4.4 Conclusions

Mitf and the novel putative downstream regulated genes presented here will provide new avenues to dissect how B cells interact with their environment, perceive, and interpret signals, and in turn program a response that controls activation and differentiation. From this perspective, future work will focus on how Mitf is positioned to regulate gene expression directly, and which signaling pathways activate/inactivate it in the setting of normal homeostasis, inducible protective immunity, and destructive autoimmunity.

## Data availability statement

The datasets presented in this study can be found in online repositoriesa. The names of the repository/repositories and accession number(s) can be found below: GSE252903 (GEO).

## Ethics statement

The studies involving humans were approved by the ethics committee/IRB in Kassambara et al., 2015 (13), from which the human B cell studies were reanalyzed. The human studies described therefore did not require additional review by our IRB. The studies were conducted in accordance with the local legislation and institutional requirements. Written informed consent for participation was not required from the participants or the participants’ legal guardians/next of kin in accordance with the national legislation and institutional requirements. The animal study was approved by SUNY Downstate Institutional Animal Care and Use Committee. The study was conducted in accordance with the local legislation and institutional requirements.

## Author contributions

AA: Conceptualization, Data curation, Formal analysis, Investigation, Methodology, Visualization, Writing – original draft, Writing – review & editing. ML-O: Conceptualization, Formal analysis, Investigation, Methodology, Writing – review & editing. RD: Formal analysis, Investigation, Methodology, Writing – review & editing. KA: Formal analysis, Investigation, Methodology, Visualization, Writing – review & editing. JD: Formal analysis, Investigation, Visualization, Writing – review & editing. NM: Formal analysis, Investigation, Writing – review & editing. CH: Conceptualization, Investigation, Supervision, Writing – review & editing, Methodology. EM: Investigation, Methodology, Supervision, Writing – review & editing. ES: Investigation, Methodology, Supervision, Writing – review & editing. CR: Conceptualization, Data curation, Formal analysis, Funding acquisition, Investigation, Methodology, Project administration, Resources, Supervision, Visualization, Writing – original draft, Writing – review & editing.

## Supplementary material

Supplementary Table 1 46,772 transcripts that passed filtering quality control in Sleuth with likelihood ratio q values, pairwise wald test q values, transcripts per million data, and pairwise log2 fold change data. (See separately included data file). Raw datasets generated from this study are available on sequence read archive (GSE252903).

Supplementary Table 2 Summary of mouse models in RNA-Seq experiments. Summarizes MiT family inhibition, cell type affected, and previously published features of each model, including TDN-B, TDN-BT, Mitfmi-vga9/mi-vga9, Mitfmi-vga9/+. Summary of the mi/mi chimera from Lin et al., 2004 is also included.

Supplementary Table 3 9303 transcripts identified in Kassambara et al., 2015 (13) aligned with both, quantification results from Kassambara et al., 2015 (13) and the present study. While unmanipulated Log2 GC robust multi-array average quantification data are presented in and **Supplementary Figure 8**, **Supplementary Table 3** includes normalized data from **Supplementary Tables** presented in Kassambara et al., 2015 (13). (See separately included data file).

Supplementary Figure 1 Immunocytochemistry of TDN-B Cells. **(A, B)** Immunocytochemistry staining to detect total nucleated splenocytes (DAPI), TDN intracellular expression (Hemagglutinin(HA)-TDN) and B cells (B220). Representative of at least three experiments. **(B)** The intensity of HA staining, minus background, in B cells (DAPI and B220 stained) and non-B cells (only DAPI stained) in TDN-B splenocytes. Bars show mean with the SEM of 20-40 cells per group. **(C, D)** Frequencies of lymphocytes and CD19+ B cells among wildtype, TDN-B, and Mitf^mi-vga9/mi-vga9^ splenocytes. 2-4 months of age. Similar findings were observed in mice at 6 months of age (data not shown). All data shown age and sex matched, male and female mice.

Supplementary Figure 2 (Relates to ). Altered lymphoid compartments and lymphocyte subpopulations in Mitf and/or MiT/TFE deficient mice. Flow cytometry markers and gating strategies of **(A)** germinal center precursor B cells, **(B)** germinal center B cells, **(C)** plasma cells, **(D)** active effector and naïve helper T cells, **(E)** mature B cells, **(F)** follicular B cells, **(G)** C-C chemokine receptor type 7 helper T cells, and (H) PD-1 helper T cells. **(I)** MFI of CD54 (ICAM-1) and **(J)** proportion of CD279 (PD-1)CD4+ splenocytes from wildtype, TDN-B, TDN-BT, and Mitf^mi-vga9/mi-vga9^. **(K)** Frequency of total (CD19+), mature, Follicular B cells of wildtype, TDN-B, and Mitf^mi-vga9/mi-vga9^ mice. 3-7 months old. Kruskal-wallis w/multiple comparisons *post-hoc*, error bars indicate SEM, *p<0.05. All data shown age and sex matched, male and female mice.

Supplementary Figure 3 (Relates to ). Flow cytometric evaluation of bone marrow B lymphocyte developmental stages. **(A)** Gating scheme of B cell development stages. **(B)** Frequencies of B cell stages in wildtype, TDN-B, and Mitf^mi-vga9/mi-vga9^ mice. 3-5 months old. Kruskal-wallis w/multiple comparisons *post-hoc*, error bars indicate SEM, *p<0.05. All data shown age and sex matched, male and female mice.

Supplementary Figure 4 (Relates to ). Flow cytometric evaluation of splenic B lymphocyte developmental stages. **(A)** Gating scheme of B cell development stages **(B)** Frequencies of B cell subpopulations in wildtype, TDN-B, and Mitf^mi-vga9/mi-vga9^ mice. 4-6 months old. Kruskal-wallis w/multiple comparisons *post-hoc*, error bars indicate SEM, *p<0.05. All data shown age and sex matched, male and female mice.

Supplementary Figure 5 Venn diagram summary of phenotypic changes observed in TDN-B and Mitf^mi-vga9/mi-vga9^ mice. Left are abnormalities in TDN-B mice alone. Right are abnormalities in Mitf^mi-vga9/mi-vga9^ mice alone, and in the middle are shared abnormalities.

Supplementary Figure 6 (Relates to ). **(A)** Examples of spleen and lymph nodes from Fas*^lpr/lpr^*, and TDN-B.Fas*^lpr/lpr^* mice. **(B)** Spleen and lymph node lymphocyte and CD19+ frequencies in Fas*^lpr/lpr^*, and TDN-B.Fas*^lpr/lpr^* mice. % lymphocyte represents % of lymphocytes gated out of total live cells and %CD19 represents percent of B cells within lymphocyte gate. **(C)** Anti-chromatin serum titers in Fas^+/lpr^, Fas*^lpr/lpr^*, and TDN-B.Fas*^lpr/lpr^* mice. 6 months old. All data shown age and sex matched, male and female mice.

Supplementary Figure 7 (Relates to ). Viability, proliferation, and apoptosis evaluation in B cells with Mitf and/or MiT/TFE family functional impairment. **(A, B)** Percent change of live (AnnexinV-Live/DeadStainlow), early apoptosis (AnnexinV+Live/DeadStainlow), and late apoptosis/dead (AnnexinV+Live/DeadStainHigh) purified B cell frequencies with and without LPS stimulation. **(C)** Tetrazolium (MTS) proliferation assay in wildtype, TDN-B, and Mitf^mi-vga9/mi-vga9^ purified B cells. Hours indicate time in MTS-proliferation assay post-48 hours of culture with LPS or anti-IgM + anti-CD40 stimulation. **(D)** Frequencies of CD19+CD95+ in ex-vivo splenocytes of wildtype, TDN-B, Mitf^mi-vga9/mi-vga9^ mice. 3-5 months old. All data shown age and sex matched, male and female mice.

Supplementary Figure 8 Venn diagram summary of gene transcription changes observed in TDN-B and Mitf^mi-vga9/mi-vga9^ mice. Left are abnormalities in TDN-B mice alone. Right are abnormalities in Mitf^mi-vga9/mi-vga9^ mice alone, and in the middle are shared abnormalities. *indicates transcript differential expression also appreciated in TDN-B/T B cells.

Supplementary Figure 9 Log2 GC robust multi-array average quantification identified as significantly differentially expressed across eight mature B cell stages and plasma cells. Clockwise, Mybl2, Mef2b, Foxm1, Stat1, Socs6, E2f7, Irf4, and Mybl1. Adapted from Kassambara et al., 2015 (13).
