# Supplementary material for: Mitf regulates gene expression networks implicated in B cell homeostasis, germinal center responses, and tolerance
Source: Front Immunol. 2024 Feb 20;15:1339325. doi: 10.3389/fimmu.2024.1339325 (PMC10912573; doi:10.3389/fimmu.2024.1339325)
Supplement: Supplementary Table 2 — Summary of mouse models in RNA-Seq experiments. Summarizes MiT family inhibition, cell type affected, and previously published features of each model, including TDN-B, TDN-BT, Mitfmi-vga9/mi-vga9, Mitfmi-vga9/+. Summary of the mi/mi chimera from Lin et al., 2004 is also included. [file DataSheet_1.docx]

Mitf regulates gene expression networks implicated in B cell homeostasis, germinal center responses, and tolerance

Abhimanyu Amarnani^1,2,3,†^, Maria Lopez-Ocasio^1,4,†^, Ramile Dilshat^5^, Kamala Anumukonda^1,7^, Jonathan Davila^2,8^, Nikita Malakhov^2,9^, Chongmin Huan^1^, Erna Magnusdottir^6^, Eirikur Steingrimsson^5^, Christopher Roman^1,2*^

^1^Program in Molecular and Cellular Biology, School of Graduate Studies, ^2^School of Medicine, SUNY Downstate Health Sciences University, Brooklyn, NY, USA

^3^Department of Medicine, Division of Rheumatology, New York University Langone Health, New York, NY, USA

^4^National Heart, Lung, and Blood Institute, National Institutes of Health, Bethesda, MD, USA.

^5^Department of Biochemistry and Molecular Biology, ^6^Department of Anatomy, Faculty of Medicine, Biomedical Center, University of Iceland, Reykjavik, Iceland.

^7^Anuko Tech Inc, Hillsborough, NJ, USA

^8^Department of Urology, Northwell Health, Staten Island, NY, USA

^9^Department of Hematology and Oncology, NYP-Weill Cornell Medical Center, New York, NY, USA
 ^†^These authors contributed equally to this work

*** Correspondence:**
Christopher.roman@downstate.edu

Keywords: B lymphocyte, tolerance, germinal center, transcriptome, *microphthalmia* transcription factor, type-I-interferon

Supplemental Figures: 9

Supplemental Tables: 3


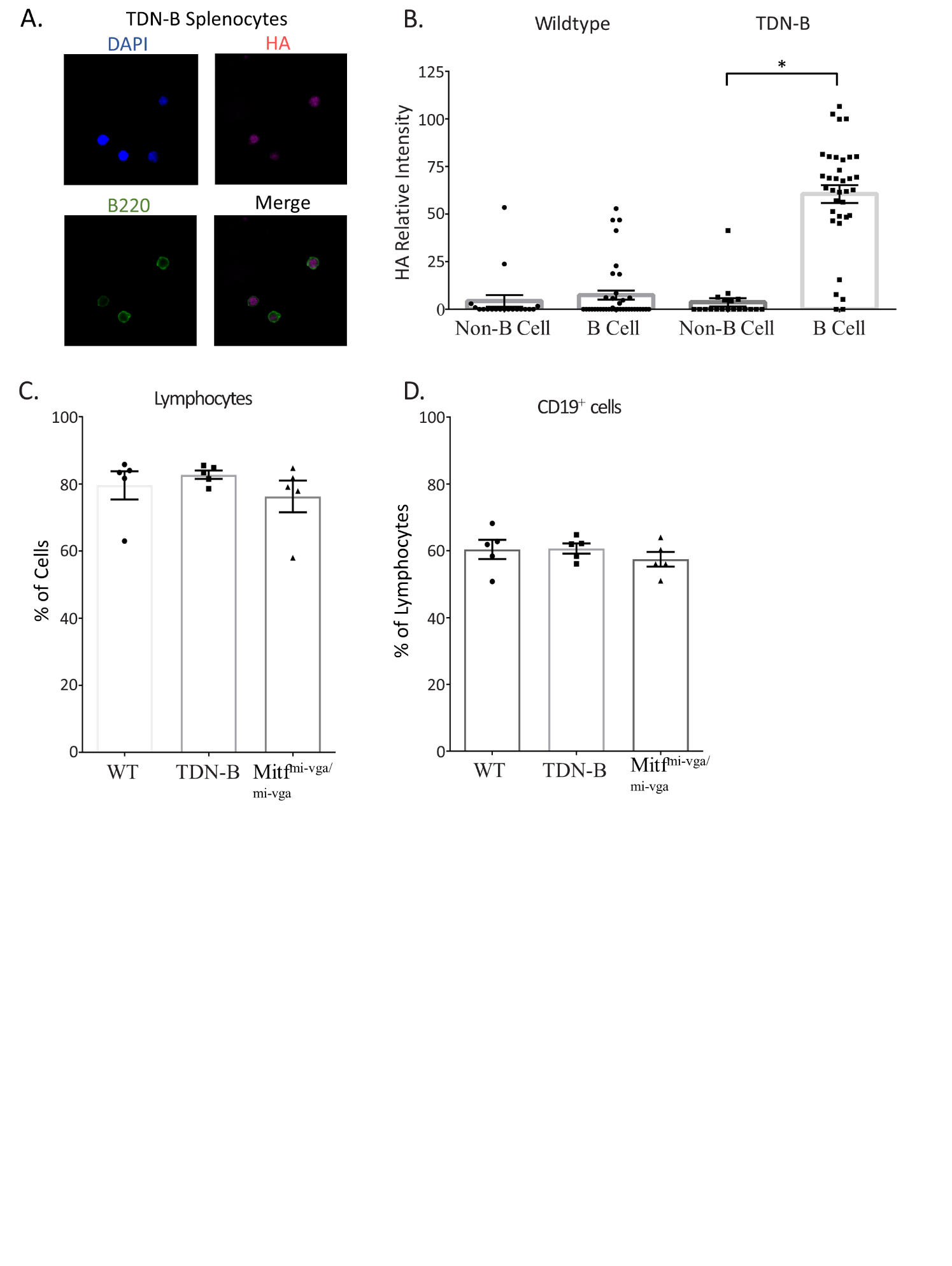


Supplemental Figure 1. Immunocytochemistry of TDN-B Cells. (A, B) Immunocytochemistry staining to detect total nucleated splenocytes (DAPI), TDN intracellular expression (Hemagglutinin(HA)-TDN) and B cells (B220). Representative of at least three experiments. (B) The intensity of HA staining, minus background, in B cells (DAPI and B220 stained) and non-B cells (only DAPI stained) in TDN-B splenocytes. Bars show mean with the SEM of 20-40 cells per group. (C, D) Frequencies of lymphocytes and CD19+ B cells among wildtype, TDN-B, and Mitf^mi-vga9/mi-vga9^ splenocytes. 2-4 months of age. Similar findings were observed in mice at 6 months of age (data not shown). All data shown age and sex matched, male and female mice.


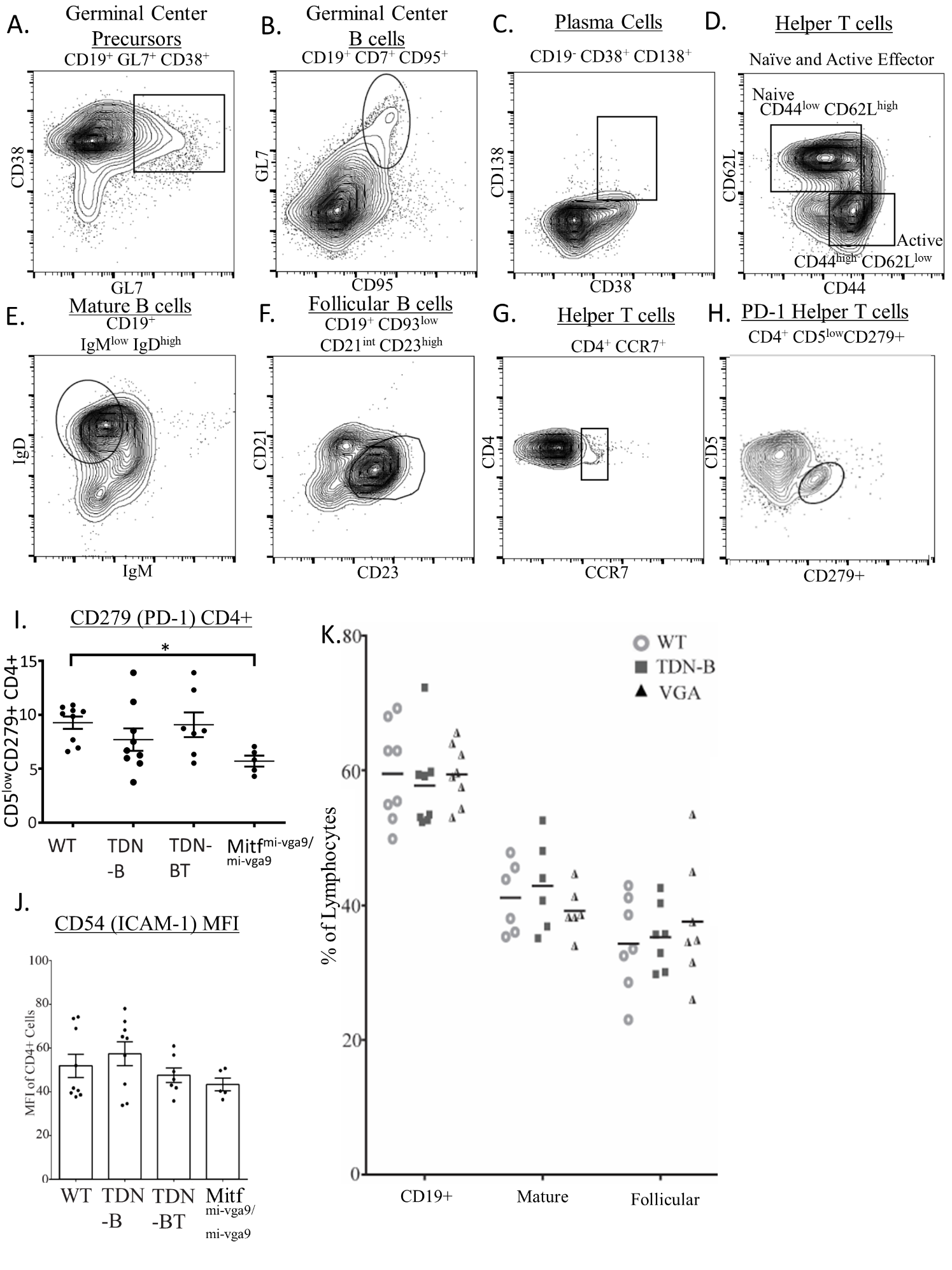


Supplemental Figure 2 (Relates to Figure 1). Altered lymphoid compartments and lymphocyte subpopulations in Mitf and/or MiT/TFE deficient mice. Flow cytometry markers and gating strategies of (A) germinal center precursor B cells, (B) germinal center B cells, (C) plasma cells, (D) active effector and naïve helper T cells, (E) mature B cells, (F) follicular B cells, (G) C-C chemokine receptor type 7 helper T cells, and (H) PD-1 helper T cells. (I) MFI of CD54 (ICAM-1) and (J) proportion of CD279 (PD-1)CD4+ splenocytes from wildtype, TDN-B, TDN-BT, and Mitf^mi-vga9/mi-vga9^. (K) Frequency of total (CD19+), mature, Follicular B cells of wildtype, TDN-B, and Mitf^mi-vga9/mi-vga9^ mice. 3-7 months old. Kruskal-wallis w/ multiple comparisons post-hoc, error bars indicate SEM, *p<0.05. All data shown age and sex matched, male and female mice.


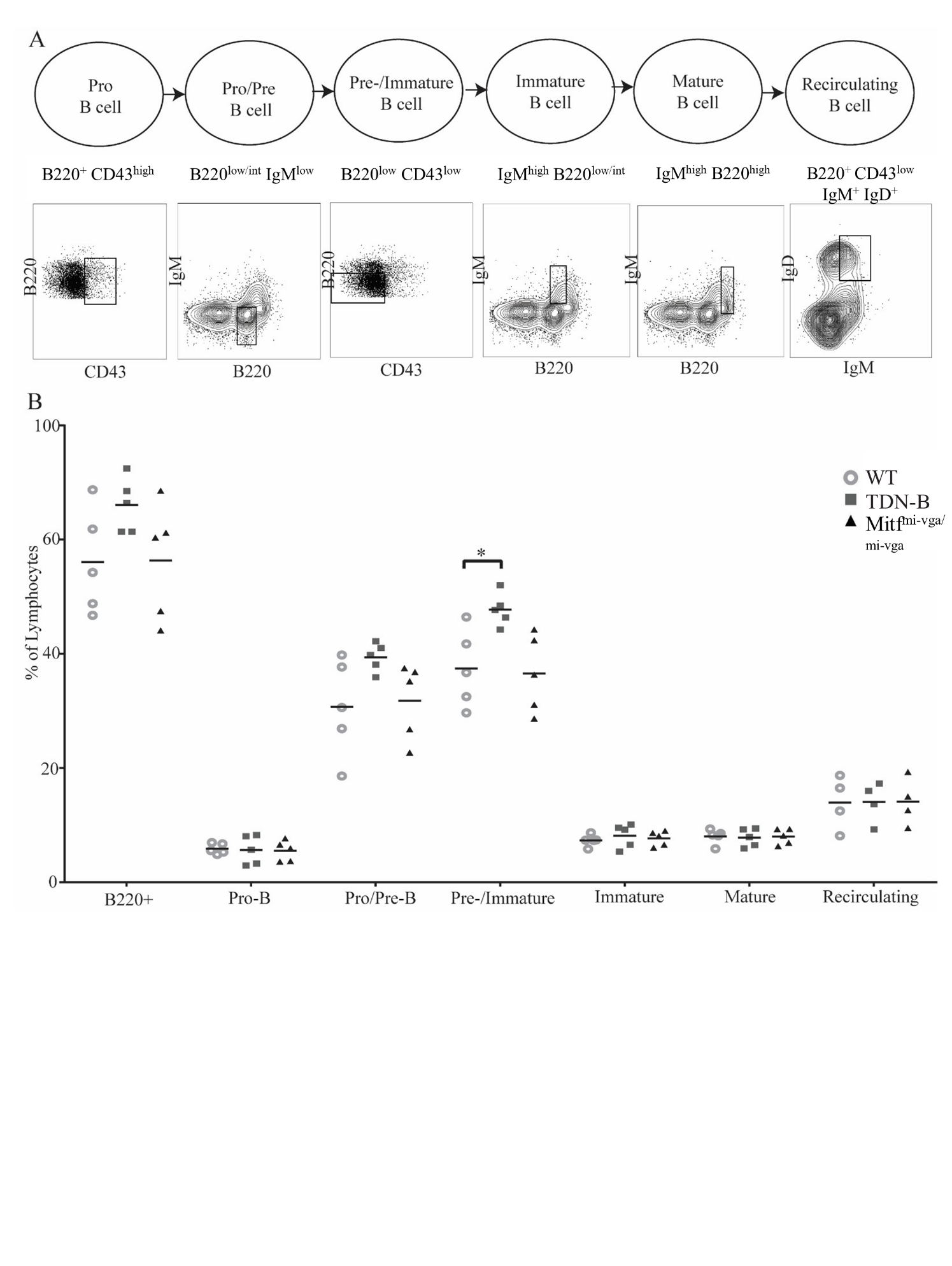


Supplemental Figure 3 (Relates to Figure 1). Flow cytometric evaluation of bone marrow B lymphocyte developmental stages. (A) Gating scheme of B cell development stages. (B) Frequencies of B cell stages in wildtype, TDN-B, and Mitf^mi-vga9/mi-vga9^ mice. 3-5 months old. Kruskal-wallis w/ multiple comparisons post-hoc, error bars indicate SEM, *p<0.05. All data shown age and sex matched, male and female mice.


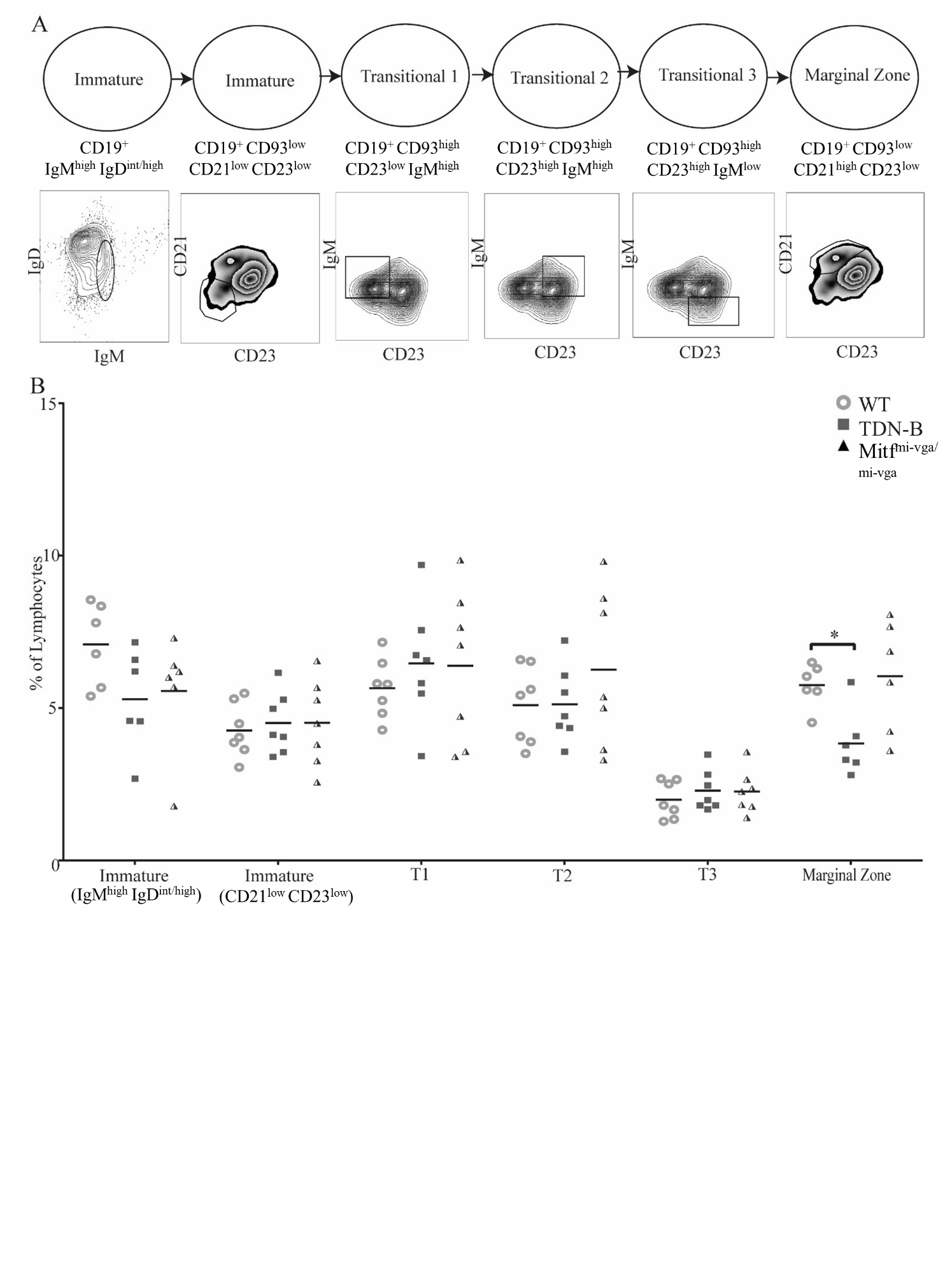


Supplemental Figure 4 (Relates to Figure 1). Flow cytometric evaluation of splenic B lymphocyte developmental stages. (A) Gating scheme of B cell development stages (B) Frequencies of B cell subpopulations in wildtype, TDN-B, and Mitf^mi-vga9/mi-vga9^ mice. 4-6 months old. Kruskal-wallis w/ multiple comparisons post-hoc, error bars indicate SEM, *p<0.05. All data shown age and sex matched, male and female mice.


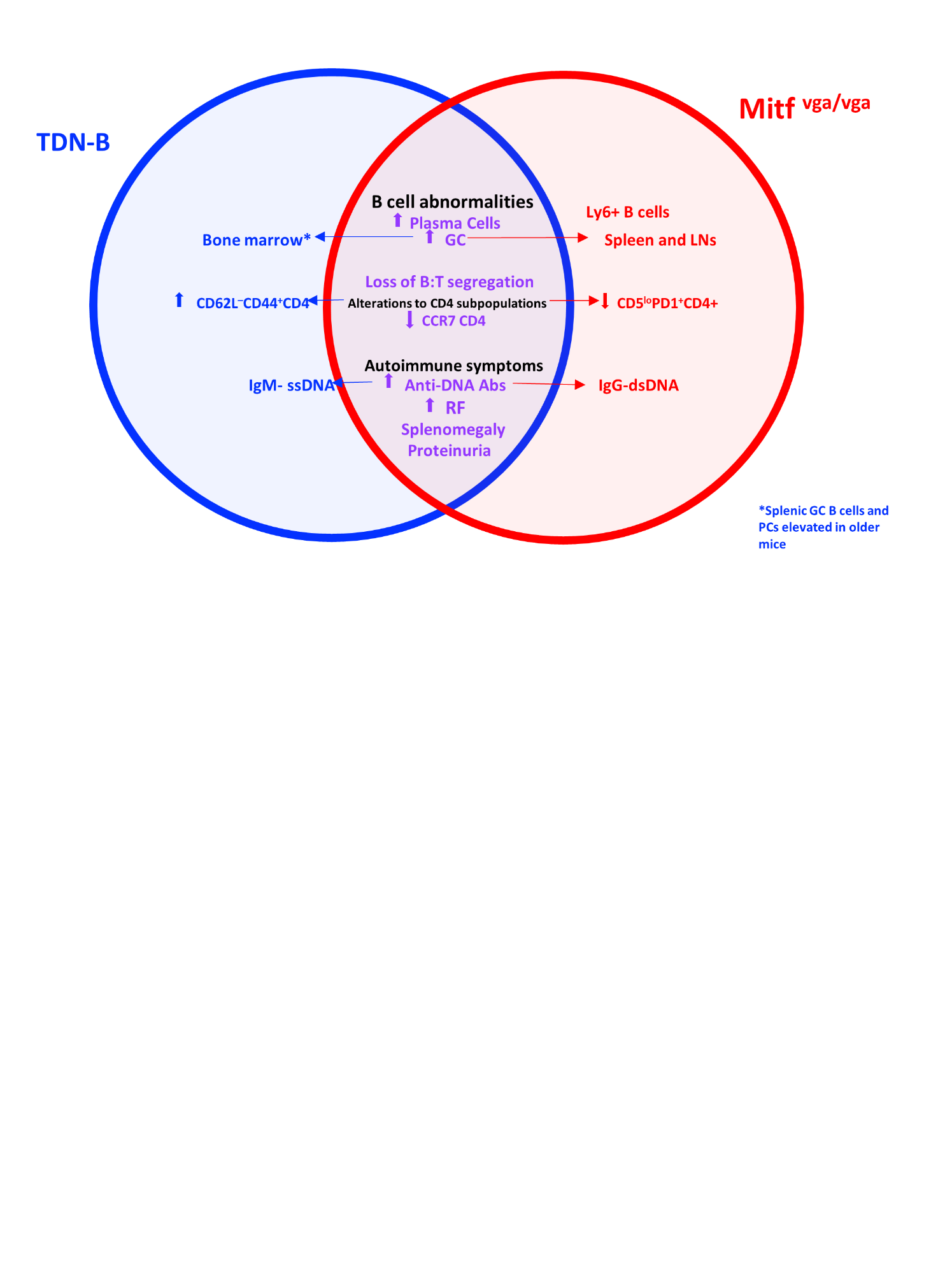


Supplemental Figure 5. Venn diagram summary of phenotypic changes observed in TDN-B and Mitf^mi-vga9/mi-vga9^ mice. Left are abnormalities in TDN-B mice alone. Right are abnormalities in Mitf^mi-vga9/mi-vga9^ mice alone, and in the middle are shared abnormalities.


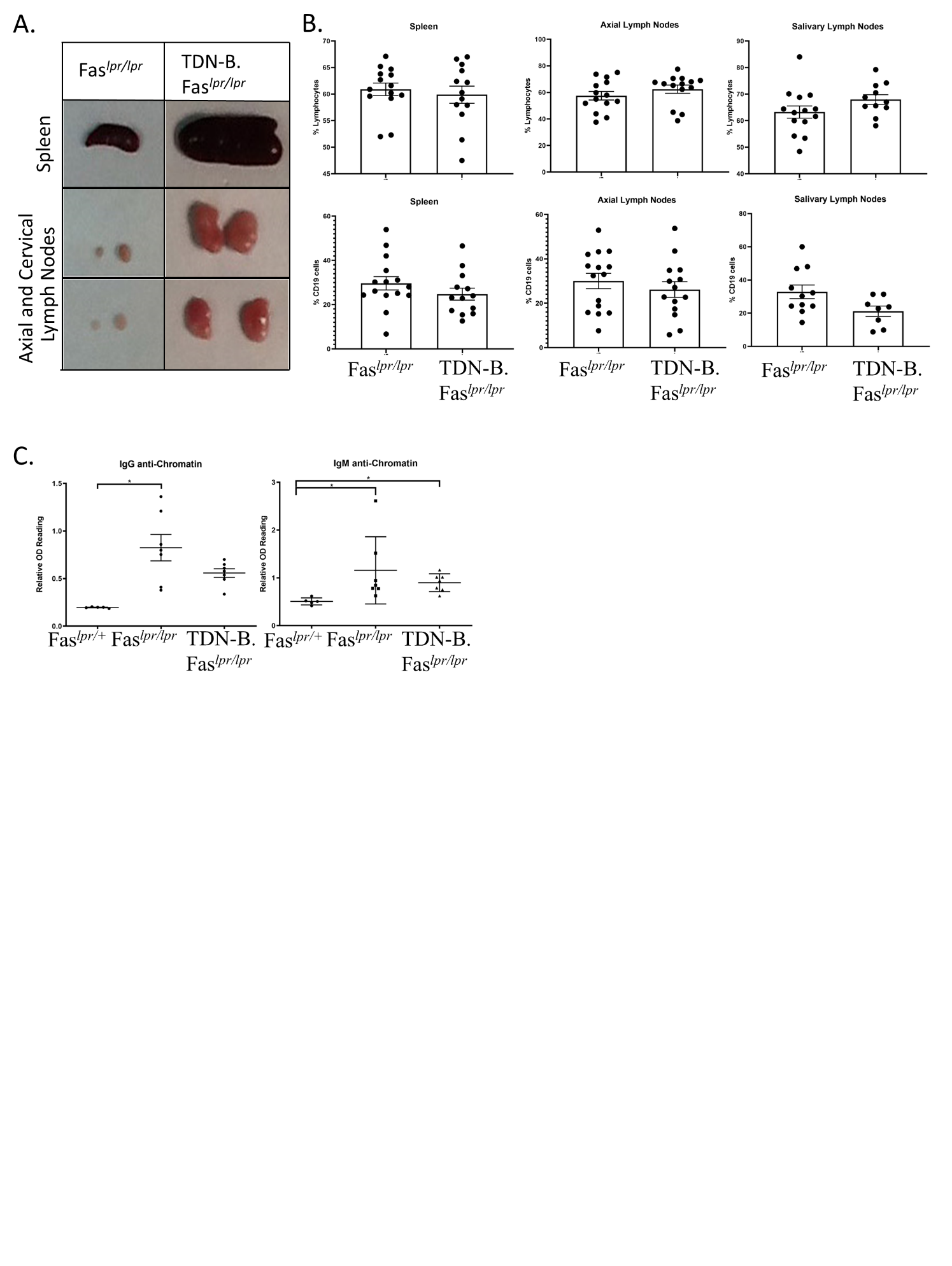


Supplemental Figure 6 (Relates to Figure 3). (A) Examples of spleen and lymph nodes from Fas*^lpr/lpr^*, and TDN-B.Fas*^lpr/lpr^* mice. (B) Spleen and lymph node lymphocyte and CD19+ frequencies in Fas*^lpr/lpr^*, and TDN-B.Fas*^lpr/lpr^* mice. % lymphocyte represents % of lymphocytes gated out of total live cells and %CD19 represents percent of B cells within lymphocyte gate. (C) Anti-chromatin serum titers in Fas^+/lpr^, Fas*^lpr/lpr^*, and TDN-B.Fas*^lpr/lpr^* mice. 6 months old. All data shown age and sex matched, male and female mice.

Supplemental Table 1. 46,772 transcripts that passed filtering quality control in Sleuth with likelihood ratio q values, pairwise wald test q values, transcripts per million data, and pairwise log2 fold change data. (See separate .xls data file). Raw datasets generated from this study are available on sequence read archive (GSE252903).

| Mouse Models | MiT Inhibition | Cell Type Affected | Previously Published features |
| --- | --- | --- | --- |
| *mi/mi* chimeras  Lin et al., 2004 | MiT family | Hematopoietic cells | - Increased plasma cells, anti-DNA autoantibodies, and rheumatoid factor  - Increased Irf4 mRNA expression in-vitro - No direct Mitf binding site identified on Irf4 promoter through EMSA |
| TDN-B | MiT family | B cells |  |
| TDN-BT | MiT family | B and T cells | - CD40L impairment with MiT inhibition in T cells Huan et al., 2006 |
| Mitf^mi-vga9/mi-vga9^ | Recessive Mitf-null mutation, with no effect on other MiT family members | All cells | - Albino mice - No previously studied or described lymphocyte abnormalities (citation) |
| Mitf^mi-vga9/+^ | Heterozygate Recessive Mitf-null mutation | All cells | -Increased type I interferon signature in melanoblasts (citation) |

Supplemental table 2. Summary of mouse models in RNA-Seq experiments. Summarizes MiT family inhibition, cell type affected, and previously published features of each model, including TDN-B, TDN-BT, Mitfmi-vga9/mi-vga9, Mitfmi-vga9/+. Summary of the mi/mi chimera from Lin et. al. 2004 is also included.


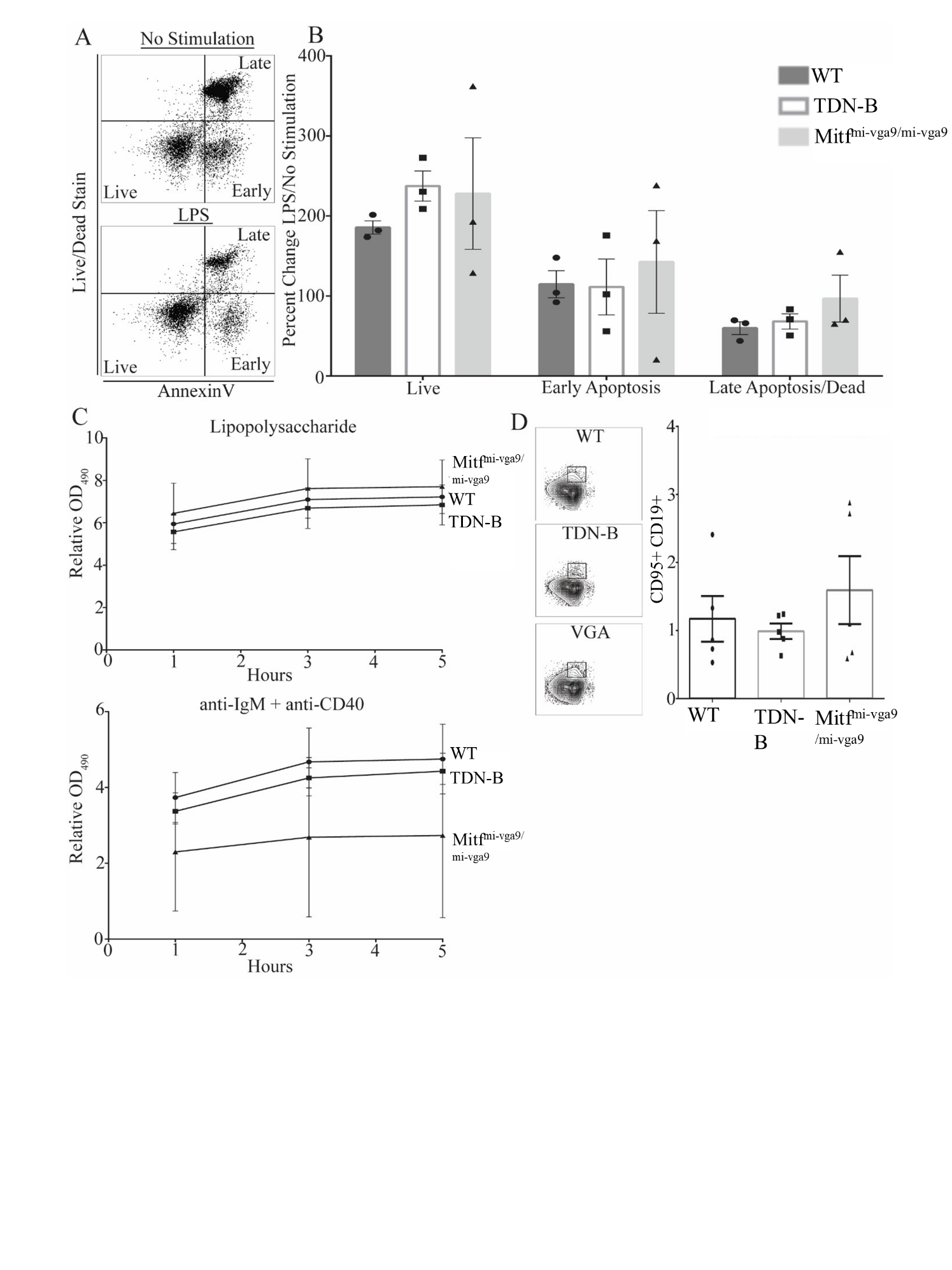
Supplemental Figure 7 (Relates to Figure 6). Viability, proliferation, and apoptosis evaluation in B cells with Mitf and/or MiT/TFE family functional impairment. (A, B) Percent change of live (AnnexinV-Live/DeadStainlow), early apoptosis (AnnexinV+Live/DeadStainlow), and late apoptosis/dead (AnnexinV+Live/DeadStainHigh) purified B cell frequencies with and without LPS stimulation. (C) Tetrazolium (MTS) proliferation assay in wildtype, TDN-B, and Mitf^mi-vga9/mi-vga9^ purified B cells. Hours indicate time in MTS-proliferation assay post-48 hours of culture with LPS or anti-IgM + anti-CD40 stimulation. (D) Frequencies of CD19+CD95+ in ex-vivo splenocytes of wildtype, TDN-B, Mitf^mi-vga9/mi-vga9^ mice. 3-5 months old. All data shown age and sex matched, male and female mice.


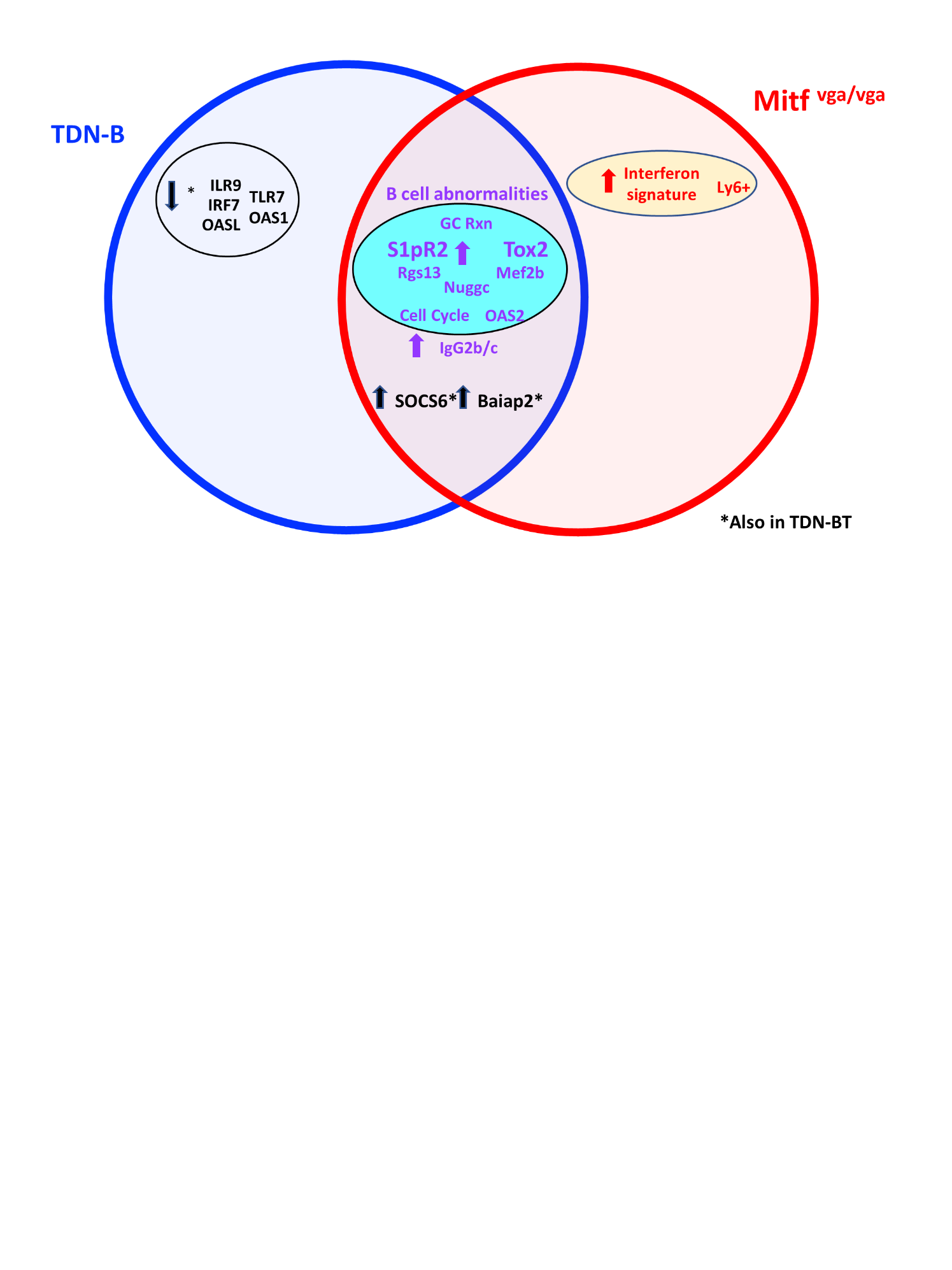
 Supplemental Figure 8. Venn diagram summary of gene transcription changes observed in TDN-B and Mitf^mi-vga9/mi-vga9^ mice. Left are abnormalities in TDN-B mice alone. Right are abnormalities in Mitf^mi-vga9/mi-vga9^ mice alone, and in the middle are shared abnormalities. *indicates transcript differential expression also appreciated in TDN-B/T B cells.


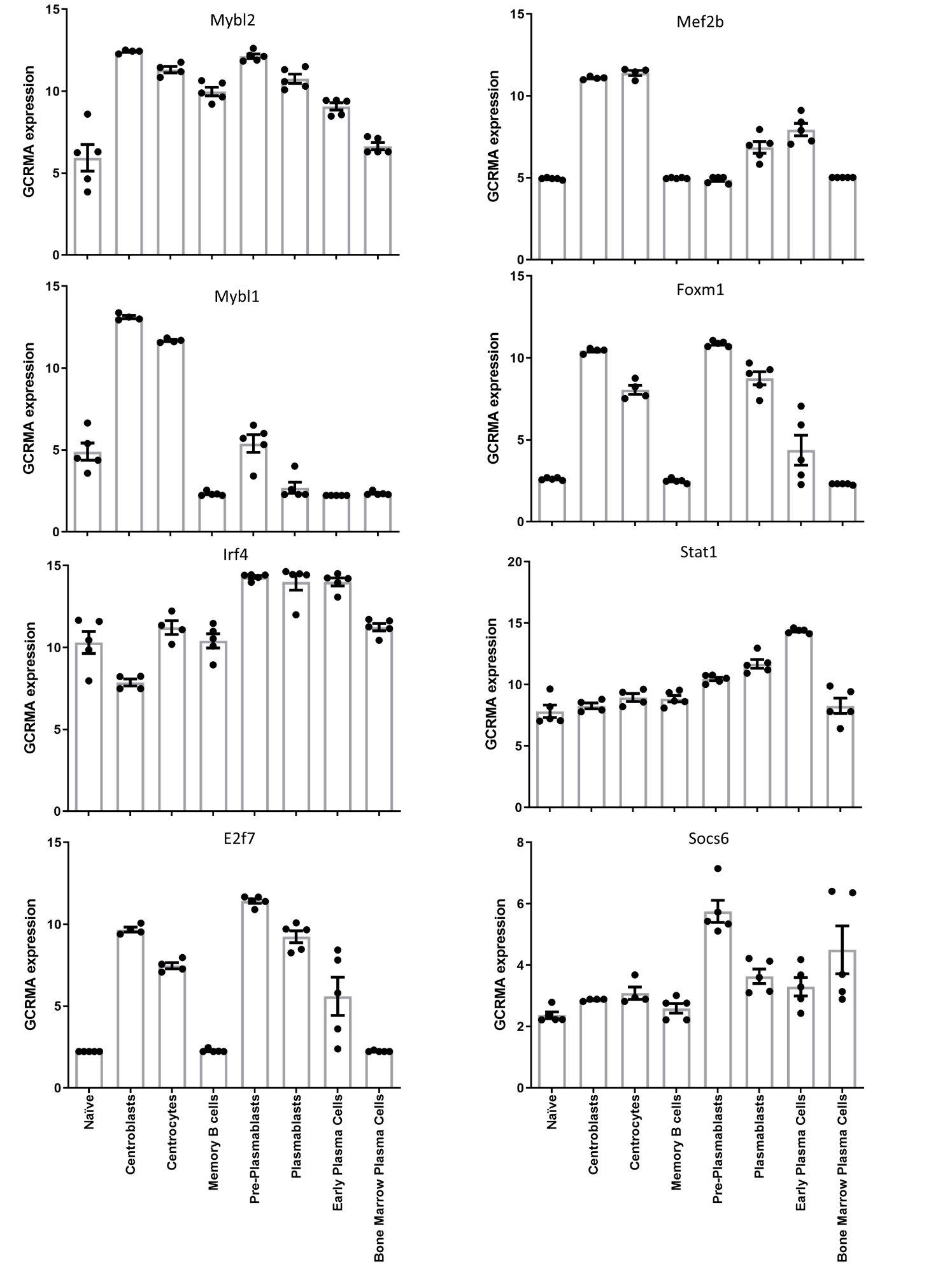


Supplemental Figure 9. Log2 GC robust multi-array average quantification identified as significantly differentially expressed across eight mature B cell stages and plasma cells. Clockwise, Mybl2, Mef2b, Foxm1, Stat1, Socs6, E2f7, Irf4, and Mybl1. Adapted from Kassambara et al., 2015.

Supplemental Table 3. 9303 transcripts identified in Kassambara et al., 2015 aligned with both, quantification results from Kassambara et al., 2015 and the present study. While unmanipulated Log2 GC robust multi-array average quantification data are presented in Figure 7 and Supplemental Figure 8, supplemental table 3 includes normalized data from supplemental tables presented in Kassambara et al., 2015. (See separately included .xls data file).
